# The effects of communicating uncertainty on public trust in facts and numbers

**DOI:** 10.1073/pnas.1913678117

**Published:** 2020-03-23

**Authors:** Anne Marthe van der Bles, Sander van der Linden, Alexandra L. J. Freeman, David J. Spiegelhalter

**Affiliations:** ^a^Winton Centre for Risk and Evidence Communication, University of Cambridge, Cambridge CB3 0WA, United Kingdom;; ^b^Department of Pure Mathematics and Mathematical Statistics, University of Cambridge, Cambridge CB3 0WA, United Kingdom;; ^c^Department of Social Psychology, University of Groningen, 19712 TS Groningen, The Netherlands;; ^d^Cambridge Social Decision-Making Lab, Department of Psychology, University of Cambridge, Cambridge CB2 3RQ, United Kingdom

**Keywords:** communication, uncertainty, trust, posttruth, contested

## Abstract

Uncertainty is inherent to our knowledge about the state of the world yet often not communicated alongside scientific facts and numbers. In the “posttruth” era where facts are increasingly contested, a common assumption is that communicating uncertainty will reduce public trust. However, a lack of systematic research makes it difficult to evaluate such claims. We conducted five experiments—including one preregistered replication with a national sample and one field experiment on the *BBC News* website (total *n* = 5,780)—to examine whether communicating epistemic uncertainty about facts across different topics (e.g., global warming, immigration), formats (verbal vs. numeric), and magnitudes (high vs. low) influences public trust. Results show that whereas people do perceive greater uncertainty when it is communicated, we observed only a small decrease in trust in numbers and trustworthiness of the source, and mostly for verbal uncertainty communication. These results could help reassure all communicators of facts and science that they can be more open and transparent about the limits of human knowledge.

## Significance

Does openly communicating uncertainty around facts and numbers necessarily undermine audiences’ trust in the facts, or the communicators? Despite concerns among scientists, experts, and journalists, this has not been studied extensively. In four experiments and one field experiment on the *BBC News* website, words and numerical ranges were used to communicate uncertainty in news article-like texts. The texts included contested topics such as climate change and immigration statistics. While people’s prior beliefs about topics influenced their trust in the facts, they did not influence how people responded to the uncertainty being communicated. Communicating uncertainty numerically only exerted a minor effect on trust. Knowing this should allow academics and science communicators to be more transparent about the limits of human knowledge.

Our knowledge is inherently uncertain. The process by which we gather information about the state of the world is characterized by assumptions, limitations, extrapolations, and generalizations, which brings imprecisions and uncertainties to the facts, numbers, and scientific hypotheses that express our understanding of the world around us. However, despite the fact that scientists and other producers of knowledge are usually well-aware of the uncertainties around their findings, these are often not communicated clearly to the public and other key stakeholders (1). This lack of transparency could potentially compromise important decisions people make based on scientific or statistical evidence, from personal medical decisions to government policies.

Recent societal developments do not seem to encourage more openness about uncertainty: It has been suggested that we are living in a “posttruth” era in which facts, evidence, and experts are deeply mistrusted (2). Cross-national survey studies suggest that in many countries, trust in institutions and governments is in decline (3–5). Although the underlying causes of changes in trust are likely to be complex and varied, it has been suggested that one way to potentially repair and restore public trust in science, evidence, and official statistics is to be more open and transparent about scientific uncertainty (2). However, it is often assumed that communicating uncertainty transparently will invite criticism, can signal incompetence, or even decrease public trust in science (1, 6–8). In fact, as summarized by the National Academies of Sciences, Engineering, and Medicine report on science communication, “as a rule, people dislike uncertainty [...] people may attribute uncertainty to poor science [… and] in some cases, communicating uncertainty can diminish perceived scientific authority” (ref. 7, pp. 27–28). For example, research by Johnson and Slovic (9) found that for some respondents, uncertainty “evoked doubt about agency trustworthiness” (p. 490), and that “despite the general sense of honesty evoked [by uncertainty] … this did not seem to offset concerns about the agency’s competence” (p. 491). In fact, partly for these reasons, Fischhoff (1) notes that scientists may be reluctant to discuss the uncertainties of their work. This hesitation spans across domains: For example, journalists find it difficult to communicate scientific uncertainty and regularly choose to ignore it altogether (10, 11). Physicians are reluctant to communicate uncertainty about evidence to patients (12), fearing that the complexity of uncertainty may overwhelm and confuse patients (13, 14). Osman et al. (15) even go as far as to argue explicitly that “the drive to increase transparency on uncertainty of the scientific process specifically does more harm than good” (p. 131).

At the same time, many organizations that produce and communicate evidence to the public, such as the European Food Safety Authority, have committed themselves to openness and transparency about their (scientific) work, which includes communicating uncertainties around evidence (16–19). These attempts have not gone without criticism and discussion about the potential impacts on public opinion (15, 20). What exactly do we know about the effects of communicating uncertainty around facts, numbers, and science to the public?

Prior research has distinguished between two kinds of uncertainty: epistemic uncertainty about the past and present state of the world that arises because of what we do not know but could know in theory (e.g., uncertainty due to limitations of the sample or methodology) vs. uncertainty about the future that arises because we cannot know (i.e., randomness, chance; we cannot know for certain what will happen tomorrow) (21). Although uncertainty about the future is a widely acknowledged aspect of forecasts and predictions, epistemic uncertainty about the past and present is equally important yet often overlooked in communication. It is the uncertainty around the decrease in unemployment in the United Kingdom (e.g., estimated at a decrease of 119,000 people from January to March 2019 compared to a year earlier, with a 95% CI of ±96,000) (22); or uncertainty around the number of people who attended US President Trump’s inauguration, which he famously claimed “had the biggest audience in the history of inaugural speeches” (23) (attendance is estimated to have been between 300,000 and 600,000) (24). Psychological research suggests that people intuitively distinguish between these two kinds of uncertainty (25, 26).

How people react to aleatoric uncertainty about the future has been relatively well studied. A large literature indicates that people are generally averse to uncertainty when making decisions about the future—a psychological tendency known as ambiguity aversion (27). Moreover, the relatively large body of research on the interpretation of verbal expressions of uncertainty such as “likely” or “unlikely” shows that there is considerable variability between people in how they interpret these words, creating problems for effective communication (28–31). It has therefore been suggested that communicating uncertainty numerically might be preferable; yet several studies found that numerical uncertainty might suffer from its own interpretation issues (32–34). To illustrate, recent research has found that motivated cognition can have an impact on probabilistic reasoning: Prior beliefs about climate change or gun laws in the United States influenced people’s interpretation of the distribution underlying ambiguous numerical ranges about these issues (35).

However, much less research has specifically focused on epistemic uncertainty. In fact, a recent review concluded that empirical evidence about the psychological effects—positive or negative—of communicating epistemic uncertainty about facts and numbers is limited and scattered with mixed findings (21). For example, research by Johnson and Slovic (9, 33, 36) suggests that communicating uncertainty via numerical ranges signaled honesty and competency for some of their participants, but dishonesty and incompetency for others. Other studies, for example, in the context of cancer risk or scientific evidence about climate change and genetically modified organisms, found that communicating uncertainty around estimates did not seem to affect people’s scientific beliefs or credibility judgments (8, 37, 38).

Given these mixed findings, the present research program aims to address this gap in the literature and is one of the first to examine the effects of communicating epistemic uncertainty about facts, numbers, and evidence on public trust. We examine the effects of communicating uncertainty around numbers—including contested numbers—that are communicated routinely in the media, such as the unemployment rate and migration statistics. Specifically, we draw on a recent theoretical review (21), which suggests that research on communicating epistemic uncertainty should consider its effects on three key conceptual dimensions, namely, 1) cognition (how people perceive and understand uncertainty), 2) emotion (how people feel about the uncertainty), and 3) trustworthiness (the extent to which people trust the information). Following recent work on source credibility in science communication (39), we further consider and conceptually distinguish two key forms of trust: people’s trust in the numbers themselves and people’s trust in the “source” of these numbers, for example, in the organization producing the statistics. From a purely statistical point of view, variability around a central estimate signals that the estimate is more uncertain; thus, when uncertainty is taken into account, a logical conclusion would be that the central estimate is less informative and reliable. However, communicating uncertainty when it exists can also be seen as part of an organization’s goal to be open and transparent, which could foster perceived trustworthiness (40). Given limited and mixed prior findings, we set out to systematically examine how communicating uncertainty about a range of facts influences public trust in both numbers and their sources in four large and diverse online experiments (combined *n* = 4,249) and one field experiment on the *BBC News* website (*n* = 1,531). We were particularly interested in comparing the effects of communicating both verbal and numerical uncertainty around a (contested) numeric estimate on perceived uncertainty (cognition) and perceived trustworthiness.*

In experiment 1, 1,122 participants read a short text about one of three topics, which contained either no uncertainty (just a point estimate), uncertainty communicated as a numerical range (in addition to the point estimate), or uncertainty communicated as a verbal statement (also in addition to the point estimate). The three topics were as follows: the number of unemployed people in the United Kingdom, the number of tigers currently left in India, and the increase in the global average surface temperature between 1880 and 2010. We selected these topics to represent various “types” of numbers (large vs. small) as well as for variation in their level of “contestedness.” That is, we expected there to be different levels of variation in prior attitudes toward the topics: Whereas opinions on climate change are divided in the United Kingdom (41), we anticipated less division in opinion around the conservation of endangered animals.

In the short text about unemployment, for example, participants read that recently an official report had been published, which stated that between April and June 2017, the number of unemployed people in the United Kingdom was an estimated 1,484,000. Participants then either received no further information (control condition), or a numerical range (numerical uncertainty condition: “minimum 1,413,000 to maximum 1,555,000”), or an equivalent verbal statement (verbal uncertainty condition: “The report states that there is some uncertainty around this estimate, it could be somewhat higher or lower”). The source of the numbers was an “official report,” which was not further specified in our first experiment in order to avoid source bias.

After reading the short text, participants first indicated how the information made them feel on a feeling thermometer, then were asked to recall the number they had just read about, and subsequently were asked a series of questions about how uncertain and reliable they perceived the number to be, and how trustworthy they thought the writers of the report were. Our measures of trust were modeled after prior studies from Johnson and Slovic (9). To ensure that our findings are systematic and robust, follow-up experiments—including a preregistered replication—varied the magnitude of the uncertainty in the message, the style and format of how the uncertainty was communicated, as well as the sampling platform.

## Results

**Fig. 1.**
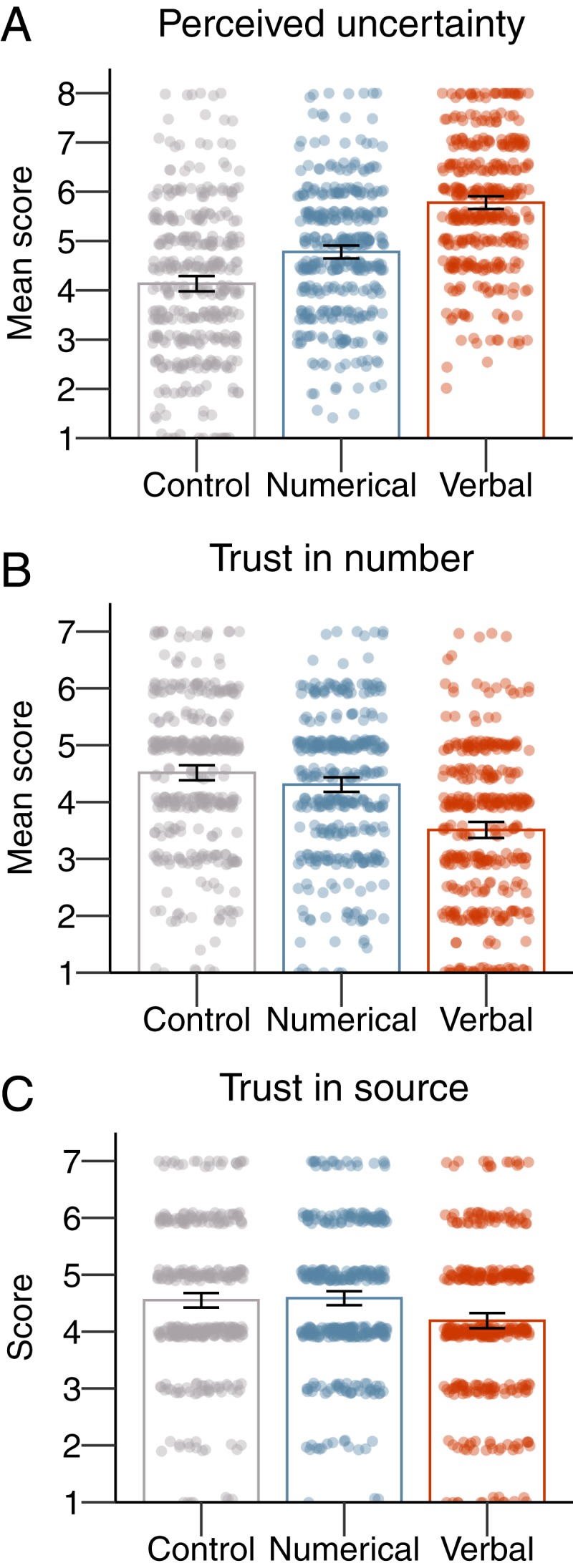
The results of experiment 1: Means per condition for perceived uncertainty (*A*), trust in numbers (*B*), and trust in the source (*C*). The error bars represent 95% CIs around the means, and jitter represents the distribution of the underlying data.

The analyses revealed no meaningful differences between topics in how people reacted to the uncertainty communication. For ease of interpretation, we therefore present the results collapsed across topics (but see *SI Appendix* for further details). The results showed that our manipulation was effective in that participants perceived the numbers to be more uncertain when uncertainty was communicated either numerically or verbally (Fig. 1*A*). An analysis of variance (ANOVA) testing the effect of uncertainty communication (control vs. numerical vs. verbal) on perceived uncertainty of the number showed a significant main effect [*F*_(2,__1119)_ = 138.67, *P* < 0.001; $\eta^{2}=20$]. All reported post hoc paired comparisons used Tukey’s honestly significant difference. People who were presented with uncertainty as a numeric range perceived the numbers to be significantly more uncertain compared to people in the control condition [M = 4.78 vs. 4.14, M_diff_ = 0.64, 95% confidence interval (CI) [0.40; 0.88], *d* = 0.45]. Participants who were presented with uncertainty as a verbal statement perceived the numbers to be significantly more uncertain than both those in the numerical (M = 5.82 vs. 4.78, M_diff_ = 1.04, 95% CI [0.80; 1.28], *d* = 0.79) and the control conditions (M = 5.82 vs. 4.14, M_diff_ = 1.68, 95% CI [1.44; 1.92], *d* = 1.17).

We also asked participants to indicate how reliable and how trustworthy they thought the numbers were; given their correlation (*r* = 0.88), scores on these two questions were combined to form a measure of “trust in numbers.” The results showed that, although our verbal phrase of uncertainty communication decreased trust in numbers, our numerical uncertainty communication did not (Fig. 1*B*). The ANOVA revealed a main effect of uncertainty communication format [*F*_(2,1119)_ = 60.96, *P* < 0.001; $\eta^{2}=0.10$]. Verbal uncertainty communication reduced people’s trust in numbers compared to the control condition (M = 3.51 vs. 4.52, M_diff_ = −1.01, 95% CI [−1.23; −0.79], *d* = 0.75) and compared to numerical uncertainty communication (M = 3.51 vs. 4.31, M_diff_ = −0.80, 95% CI [−1.03; −0.57], *d* = 0.60). Importantly, there was no significant difference in trust in numbers between the numerical uncertainty communication and control conditions (M = 4.31 vs. 4.52, M_diff_ = −0.21, 95% CI [−0.43; 0.01], *d* = 0.17).

Finally, we asked participants how trustworthy they thought “the writers of the report” were, as a measure of trust in the source. Again, we found that the verbal uncertainty communication led to a small significant decrease in people’s trust in the source, whereas the numerical uncertainty communication did not (Fig. 1*C*). The ANOVA showed a main effect of uncertainty communication format [*F*_(2,__1119)_ = 11.03, *P* < 0.001; $\eta^{2}=0.02$]. Communicating uncertainty verbally reduced participant’s trust in the source, compared to the control condition (M = 4.19 vs. 4.55, M_diff_ = −0.36, 95% CI [−0.57; −0.15], *d* = 0.28) and numerical uncertainty communication (M = 4.19 vs. 4.58, M_diff_ = −0.39, 95% CI [−0.61; −0.17], *d* = 0.31). Again, there was no significant decrease for numerical uncertainty communication compared to control (M = 4.58 vs. 4.55, M_diff_ = 0.03, 95% CI [−0.18; 0.24], *d* = 0.02).

Experiment 1 thus showed that while people did perceive uncertainty about numbers both when it was communicated numerically and verbally, only the verbal communication reduced people’s trust in the numbers and the source. In addition, the results showed no significant effect of uncertainty communication on people’s affect or mood; please see *SI Appendix* for the full results. Because we found no substantial differences between topics in people’s responses to uncertainty, experiment 2 only used the UK employment number to study whether the magnitude of the uncertainty itself can influence the psychological effects of communicating uncertainty.

### Manipulating the Magnitude of Uncertainty.

The goal of experiment 2 was twofold: first, to replicate the results from experiment 1 (for unemployment) and second, to examine whether the magnitude of the uncertainty affected people’s trust in numbers and trust in the source. This experiment followed a 1 (control condition: no uncertainty) + 2 (numerical vs. verbal communication) × 3 (lower vs. original vs. higher uncertainty) between-subject design. For numerical uncertainty communication, we presented the original 95% CI, which therefore acted as a replication of experiment 1; lower uncertainty using a range half the size (99.99% CI); or higher uncertainty using a range twice as large (68% CI) as the original CI. For verbal uncertainty communication, we presented the same baseline phrase as in experiment 1 for the “original” magnitude (“…it could be somewhat higher or lower”); less uncertainty using the phrase “slightly higher or lower”; or more uncertainty using the phrase “a lot higher or lower.” The verbal phrases were chosen to mirror the magnitude of the numerical uncertainty.

First, we analyzed the results of the “original uncertainty” levels and the control condition in experiment 2: a direct replication of experiment 1. For perceived uncertainty and trust in the number, we replicated the results of the first experiment. The analyses, all reported in detail in the *SI Appendix*, showed that participants perceived the number to be significantly more uncertain when numerical uncertainty was communicated (compared to control) and when verbal uncertainty was communicated (compared to both control and numerical uncertainty). Similarly, just as in experiment 1, participants reported less trust in the number when verbal uncertainty was communicated compared to both control and when numerical uncertainty was communicated; with no significant decrease in trust for numerical uncertainty (compared to control). However, in contrast to experiment 1 where we found that verbal uncertainty communication reduced trust in the source, experiment 2 showed no significant effect of uncertainty communication: Both numerical and verbal uncertainty communicated did not decrease people’s trust in the source, compared to control (please see *SI Appendix*, Fig. S2 *A*–*C*).

**Fig. 2.**
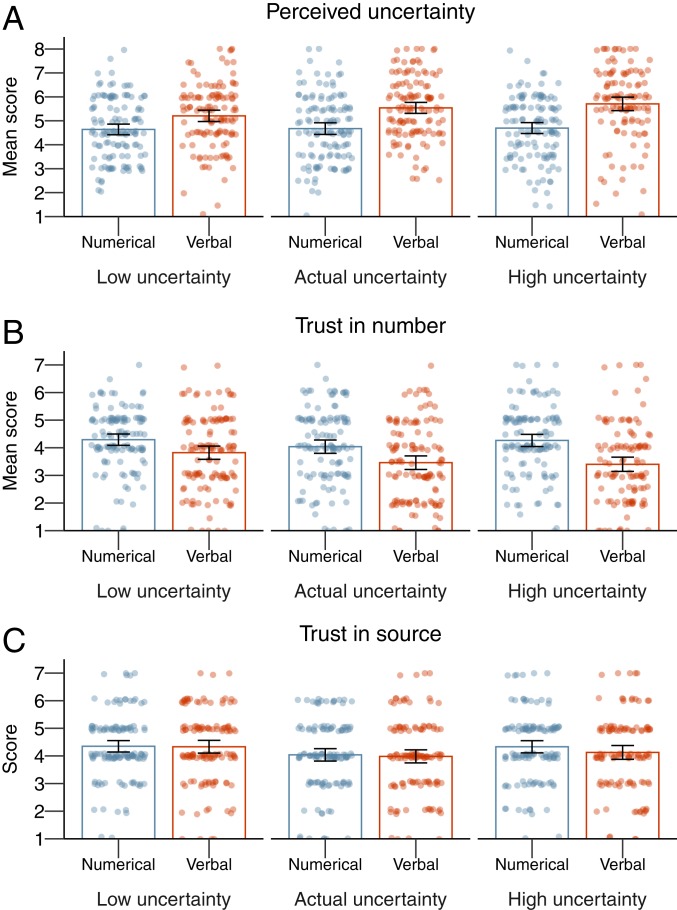
The results of experiment 2: Means per condition for perceived uncertainty (*A*), trust in numbers (*B*), and trust in the source (*C*). The error bars represent 95% CIs around the means, and jitter represents the distribution of the underlying data.

Next, we examined the effects of the magnitude of uncertainty. Somewhat surprisingly, we found that the magnitude of the communicated uncertainty did not affect people’s perceptions of the uncertainty of the numbers (Fig. 2*A*). A two-way ANOVA of format (numeric vs. verbal) and magnitude (lower vs. original vs. higher) showed a significant main effect of format [*F*_(1,__741)_ = 67.93, *P* < 0.001; $\eta_{p}^{2}=0.08$], but no significant main effect of magnitude [*F*_(2,__741)_ = 2.92, *P* = 0.055; $\eta_{p}^{2}=0.01$] nor a significant interaction [*F*_(2,__741)_ = 1.86, *P* = 0.16; $\eta_{p}^{2}=0.01$]. Regardless of magnitude, and as in experiment 1, verbal uncertainty communication led to higher levels of perceived uncertainty than numerical uncertainty communication (M = 5.50 vs. 4.68, M_diff_ = 0.83, 95% CI [0.63; 1.03], *d* = 0.60).

However, the results did show a small effect of magnitude on people’s trust in numbers (Fig. 2*B*). An ANOVA of format and magnitude showed a main effect of format [*F*_(1,__741)_ = 43.44, *P* < 0.001; $\eta_{p}^{2}=0.06$], and main effect of magnitude [*F*_(2,__741)_ = 3.63, *P* = 0.03; $\eta_{p}^{2}=0.01$], but no significant interaction [*F*_(2,__741)_ = 1.44, *P* = 0.24]. Regardless of magnitude, verbal uncertainty communication decreased trust in numbers compared to numerical communication (M = 3.56 vs. 4.20, M_diff_ = −0.64, 95% CI [−0.83; −0.45], *d* = 0.48). In addition, across formats, post hoc comparisons showed that a lower magnitude of uncertainty led to higher trust in numbers compared to the original range or phrase (M = 4.06 vs. 3.75, M_diff_ = 0.31, 95% CI [0.03; 0.58], *d* = 0.23). Both comparisons were not significantly different from higher magnitude of uncertainty (Fig. 2*B*).

For trust in the source, communication format (numerical or verbal) appeared to make no difference, but the magnitude of the uncertainty did (Fig. 2*C*). An ANOVA of format and magnitude showed no main effect of format [*F*_(1,__741)_ = 0.96, *P* = 0.33], a significant main effect of magnitude [*F*_(2,__741)_ = 4.30, *P* = 0.01; $\eta_{p}^{2}=0.01$], but no significant interaction [*F*_(2,__741)_ = 0.37, *P* = 0.69]. Regardless of format, lower magnitudes of uncertainty led to higher levels of trust in the source compared to the original range or phrase (M = 4.34 vs. 4.01, M_diff_ = 0.33, 95% CI [0.06; 0.60], *d* = 0.26), but neither were significantly different from higher magnitude of uncertainty (M = 4.23, SD = 1.31; Fig. 2*C*).

The results of experiment 2 thus suggest that magnitude, communicated without further context, did not have a strong impact on people’s reactions to uncertainty communication: It did not influence perceptions of uncertainty, and only lower magnitudes of uncertainty were related to slightly higher levels of perceived reliability of the number and trustworthiness of the source. Without further context, participants might not have been able to interpret the numerical ranges as being relatively small or large magnitudes of uncertainty, although the same is not true of the verbal conditions. Although preliminary, what we can conclude from these results is that, in the absence of further context, it appears that whether and how uncertainty is communicated is more important in determining how people respond than the magnitude of the uncertainty in question.

### Varying the Format of Uncertainty Communication.

Following these findings, we set out to systematically test the effects of additional numeric and verbal uncertainty communication formats in experiment 3, and to move toward a more realistic and better contextualized communication scenario. This experiment had eight conditions, which are presented in Table 1. The choice of formats was influenced by the formats the UK Office for National Statistics uses to communicate uncertainty around unemployment numbers, which was again the context we used for this experiment for consistency. To improve the ecological validity of the experiment, the manipulation was written as a traditional news media article and included two unemployment figures. Uncertainty was communicated in the same format around both figures.

**Table 1.** Overview of the conditions and manipulation texts of experiment 3 and 4

Format	Experiment 3	Experiment 4
Control (no uncertainty)	“Official figures from the first quarter of 2018 show that UK unemployment fell by 116,000 compared with the same period last year. […]”	“Migration figures: EU migration still adding to UK population. Official figures from last year show that there were 101,000 more people coming to the UK from the EU than leaving in 2017. This is the lowest EU net migration figure since 2013, but it means that EU migrants are still adding to the UK population. […]”
Numerical range with point estimate	…by 116,000 (range between 17,000 and 215,000)…	…101,000 (range between 68,000 and 132,000)…
Numerical range without point estimate	…by between 17,000 and 215,000…	
Numerical point estimate ±2 SEs	…by 116,000 (±99,000)…	…101,000 (±33,000)…
Verbal explicit uncertainty statement	…by 116,000 compared with the same period last year, although there is some uncertainty around this figure: It could be somewhat higher or lower. […]	…101,000 more people coming to the UK from the EU than leaving in 2017. The report states there is uncertainty around the exact figure—it could be higher or lower. […]
Verbal implicit uncertainty statement	…by 116,000 compared with the same period last year, although there is a range around this figure: could be somewhat higher or lower. […]	
Verbal uncertainty word	…by an estimated 116,000…	…around 101,000…
Mixed numerical and verbal phrase	…by an estimated 116,000 (±99,000)…	

**Fig. 3.**
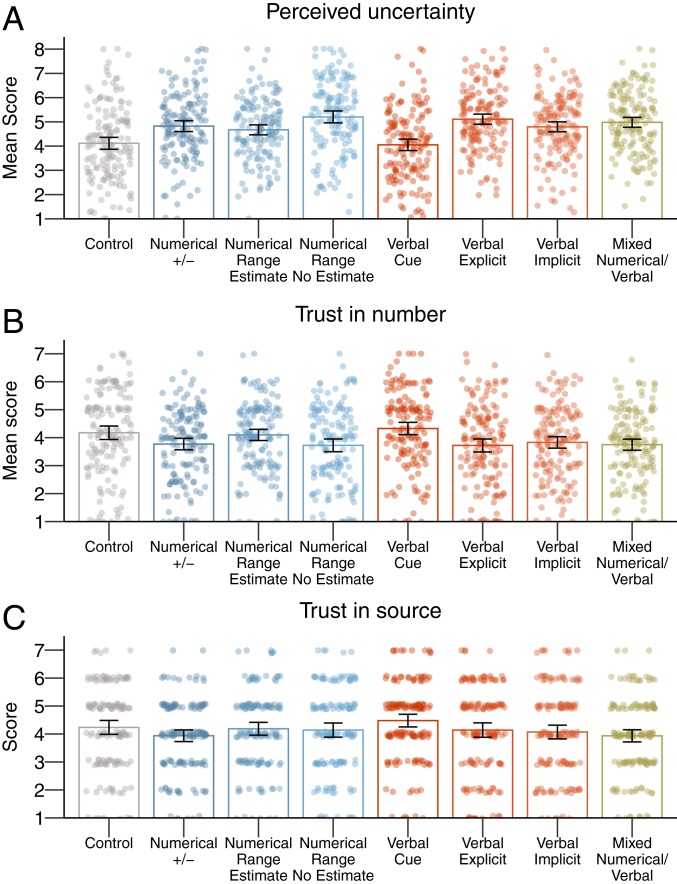
The results of experiment 3: Means per condition for perceived uncertainty (*A*), trust in numbers (*B*), and trust in the source (*C*). The error bars represent 95% CIs around the means, and jitter represents the distribution of the underlying data.

Results are presented in Fig. 3. The level of perceived uncertainty around the numbers differed between formats [Fig. 3*A*; one-way ANOVA effect of format: *F*_(7,__1192)_ = 14.43, *P* < 0.001; $\eta^{2}=0.08$]. For all formats in which uncertainty was being communicated, except one, participants perceived the numbers to be more uncertain compared to the control condition (post hoc paired comparisons*: p* values = 0.022 to <0.001; M_diff_ = 0.53 to 1.10; *d* values = 0.37 to 0.72). The exception was the condition in which only the word “estimated” had been added (M_diff_ = −0.07, *P* = 1.00). People in this condition did not perceive the numbers to be more uncertain compared to those in the control condition, indicating that only using the word “estimated” seems insufficient to communicate the existence of uncertainty around a number.

People’s trust in numbers similarly differed between formats [Fig. 3*B*; *F*_(7,__1192)_ = 5.97, *P* < 0.001; $\eta^{2}=0.03$]. Whereas for most formats, people’s trust in the numbers was significantly reduced compared to control (post hoc paired comparisons: *p* values = 0.035 to 0.011; M_diff_ = −0.47 to −0.53; *d* values = 0.35 to 0.37), this was not the case when uncertainty was communicated with the word “estimated” (M_diff_ = 0.13, 95% CI [−0.33; 0.59], *P* = 0.98), with the implicit uncertainty statement (M_diff_ = −0.37, 95% CI [−0.83; 0.08], *P* = 0.20), or with a numerical range with point estimate (M_diff_ = −0.10, 95% CI [−0.56; 0.36], *P* = 1.00).

In this experiment, we assessed trust in the source by asking people to what extent they thought that the civil servants who were responsible for the unemployment figures were trustworthy. Results are shown in Fig. 3*C*. We found that different formats did make a small difference to people’s trust in the source [one-way ANOVA: *F*_(7,__1192)_ = 2.15, *P* = 0.04; $\eta^{2}=0.01$]. However, that difference was not between the conditions in which uncertainty was communicated and the control: Across all formats, trust in the source did not differ significantly from the control condition (range M = 3.94 to 4.48 vs. M_control_ = 4.24, SD_control_ = 1.55). The difference was between specific formats: Compared to people to whom uncertainty was communicated through the word “estimated,” people to whom uncertainty was communicated in the numeric +/− format or mixed format (“estimated +/−”) perceived the source to be significantly less trustworthy (M = 4.48 vs. 3.94, M_diff_ = 0.54, 95% CI [0.02; 1.06], *d* = 0.40 and M = 4.48 vs. 3.94, M_diff_ = 0.54, 95% CI [0.03; 1.05], *d* = 0.39, respectively).

To examine the boundary conditions of the effects on trust, we also asked people to indicate how trustworthy they thought journalists who write news articles like the ones they had read were and how reliable they thought government statistics in general are; these judgements did not differ significantly for different uncertainty communication formats [*F*_(7,__1192)_ = 1.60, *P* = 0.13; $\eta^{2}=0.01$, and *F*_(7,__1192)_ = 1.60, *P* = 0.13; $\eta^{2}=0.01$, respectively].

In conclusion, then, the results of experiment 3 showed that whereas participants perceived uncertainty when uncertainty was communicated in most numeric and verbal formats, not all formats affected people’s trust in the numbers. Communicating uncertainty via a numerical range with point estimate or an implicit verbal statement did not significantly decrease trust in numbers compared to the control condition. Adding the word “estimated” also did not decrease trust, but this format apparently failed to communicate uncertainty to people. However, importantly, just as in experiment 2, none of the uncertainty communication formats decreased trust in the source compared to not communicating uncertainty: There was no impact of uncertainty communication when we asked people about the trustworthiness of the civil servants responsible for the statistics, nor for journalists who write such articles.

### Uncertainty Around Contested Numbers.

Experiments 2 and 3 were conducted in the context of UK unemployment numbers, which are generally considered not highly contested and thus might be less likely to result in changes in trust-related perceptions. We therefore conducted experiment 4 in the context of UK migration, which is a more contested issue on which public opinion is divided (42). Experiment 4 was preregistered on aspredicted.org (https://aspredicted.org/blind.php?x=d3xu67) and also conducted on a national sample of the UK population (*Methods*). Participants were first asked about their attitudes toward migration, before being randomly selected to read one of five versions of a fictitious newspaper article about migration statistics, which are presented in Table 1.

**Fig. 4.**
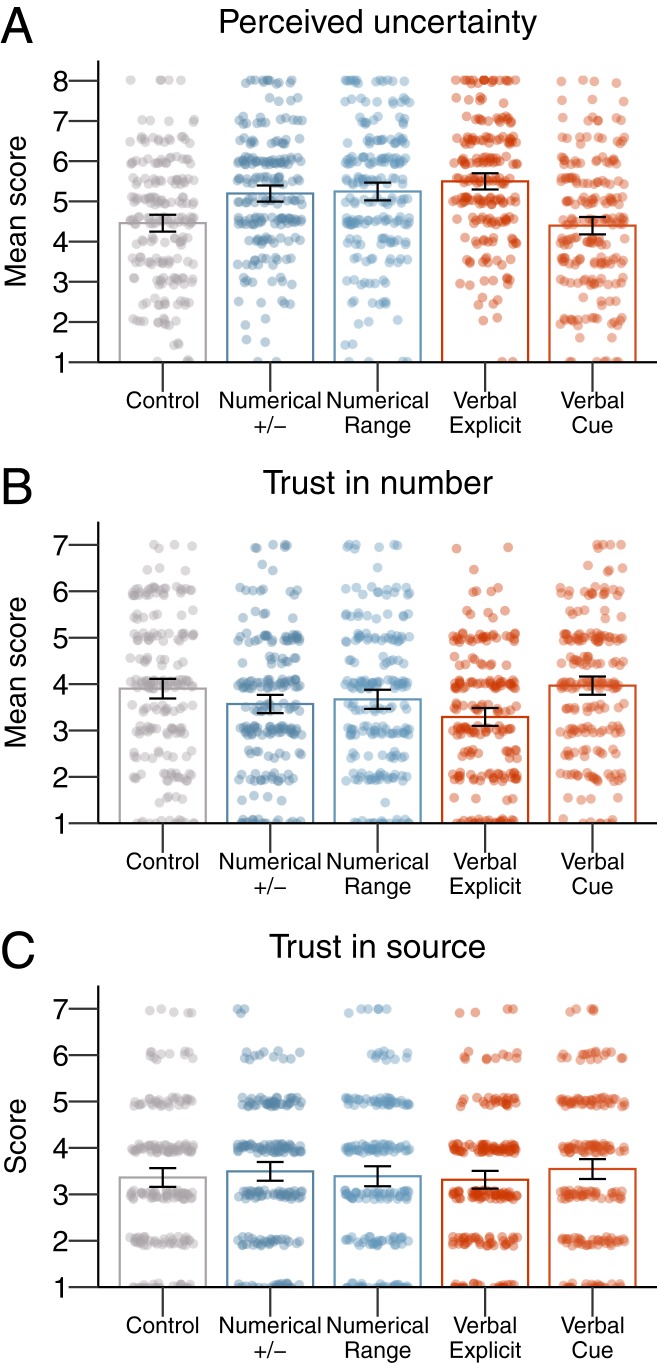
The results of experiment 4: Means per condition for perceived uncertainty (*A*), trust in numbers (*B*), and trust in the source (*C*). The error bars represent 95% CIs around the means, and the jitter represents the distribution of the underlying data.

The results of experiment 4 showed that, similar to experiment 3, participants perceived the number to be more uncertain for all communication formats compared to the control condition, except for just using the word “around” before the number [Fig. 4*A*; one-way ANOVA *F*_(4,__1045)_ = 22.11, *P* < 0.001; $\eta^{2}=0.08$]. Post hoc paired comparisons showed significant differences between the control condition vs. communicating uncertainty as a numeric range (M = 4.47 vs. 5.27, M_diff_ = −0.80, 95% CI [−1.22; −0.39], *d* = 0.50), using “+/−” (M = 4.47 vs. 5.24, M_diff_ = −0.77, 95% CI [−1.19; −0.35], *d* = 0.50), and as an explicit verbal statement (M = 4.47 vs. 5.52, M_diff_ = −1.06, 95% CI [−1.48; −0.64], *d* = 0.68).

As Fig. 4*B* shows, communication formats did affect participants’ trust in numbers [one-way ANOVA *F*_(4,__1044)_ = 7.29, *P* < 0.001; $\eta^{2}=0.03$], but this overall effect was qualified by a significant decrease in trust for the explicit verbal statement when compared to control (M = 3.28 vs. 3.90, M_diff_ = −0.62, 95% CI [−1.01; −0.23], *d* = 0.42). The numerical formats and the verbal “around” condition did not significantly reduce trust in numbers compared to control.

Furthermore, we found no effect of uncertainty communication on participants’ trust in the source, which in this experiment was assessed as perceived trustworthiness of “the civil servants responsible for the migration statistics” [*F*_(4,__1045)_ = 1.19, *P* = 0.31].

In summary, we found that whereas both numeric formats and the explicit verbal statement did communicate uncertainty around the net migration number, only the explicit verbal statement decreased perceived reliability of the number, and no format decreased participants’ perceptions of trustworthiness of the source. This pattern of results is broadly consistent with our preregistered hypotheses based on our previous three studies (see *SI Appendix* for more details).

We also asked people to what extent they thought that 1) the conclusions based on the number, 2) the news article they just read, 3) journalists who write news articles such as this one, and 4) government statistics in general were trustworthy.

This revealed that format did affect trust in the conclusions [one-way ANOVA of format: *F*_(4,__1045)_ = 3.53, *P* = 0.007; $\eta^{2}=0.01$]. This was due to the explicit verbal statement of uncertainty leading to lower trust in the conclusions than the word “around” (post hoc paired comparisons*:* M = 3.51 vs. 3.96, M_diff_ = −0.45, 95% CI [−0.84; −0.05], *d* = 0.30). All uncertainty communication formats did not differ significantly from the control condition (M = 3.90, SD = 1.46).

Second, there appeared to be a small effect of format on trust in the news article itself (one-way ANOVA of format: *F*_(4,__1042)_ = 2.43, *P* = 0.046; $\eta^{2}=0.01$), but post hoc paired comparisons did not show significant differences between formats—it was again mainly driven by a decrease for the explicit verbal statement compared to the control condition (M = 3.66 vs. 4.04, M_diff_ = −0.38, 95% CI [−0.77; 0.01], *d* = 0.26).

Third, consistent with experiment 3, participants’ trust in journalists and in government statistics in general were not significantly affected by communicating uncertainty in these different formats (one-way ANOVAs: *F*_(4,__1044)_ = 0.85, *P* = 0.50, and *F*_(4,__1045)_ = 1.73, *P* = 0.14, respectively). These additional findings suggest that communicating uncertainty verbally has an impact on the perceived reliability of the number itself and conclusions based on a number, but does not seem to impact judgements about the source(s) of numbers (civil servants or journalists), nor generalize to governmental statistics more broadly.

Given the contested nature of immigration statistics, we also explored the extent to which people’s prior attitudes toward immigration affected the results. We split our sample into two groups (based on the median = 4.33): people with negative attitudes toward immigration (mean, 1.00 to 4.00) and people with positive attitudes (mean, 4.33 to 7.00). Two-way ANOVAs (format × immigration attitude: negative vs. positive) revealed main effects of immigration attitudes on perceived uncertainty, trust in numbers, and trust in the source, but no interaction effects. People with positive attitudes toward immigration perceived less uncertainty around the numbers [*F*_(1,__1040)_ = 6.15, *P* = 0.01; $\eta_{p}^{2}=0.01$], reported more trust in the numbers [*F*_(1,__1039)_ = 33.39, *P* < 0.001; $\eta_{p}^{2}=0.03$], and more trust in the source [*F*_(1,__1040)_ = 45.22, *P* < 0.001; $\eta_{p}^{2}=0.04$] than people with negative attitudes toward immigration. However, we found no significant interaction effects between attitudes and communication format. To assess the robustness of these results, we also conducted a series of hierarchical linear regressions with a continuous interaction term. These analyses produced the same findings and are reported in the *SI Appendix*. Overall, how people responded to uncertainty communication was not affected by their prior attitudes toward immigration.

### Internal Metaanalysis.

**Fig. 5.**
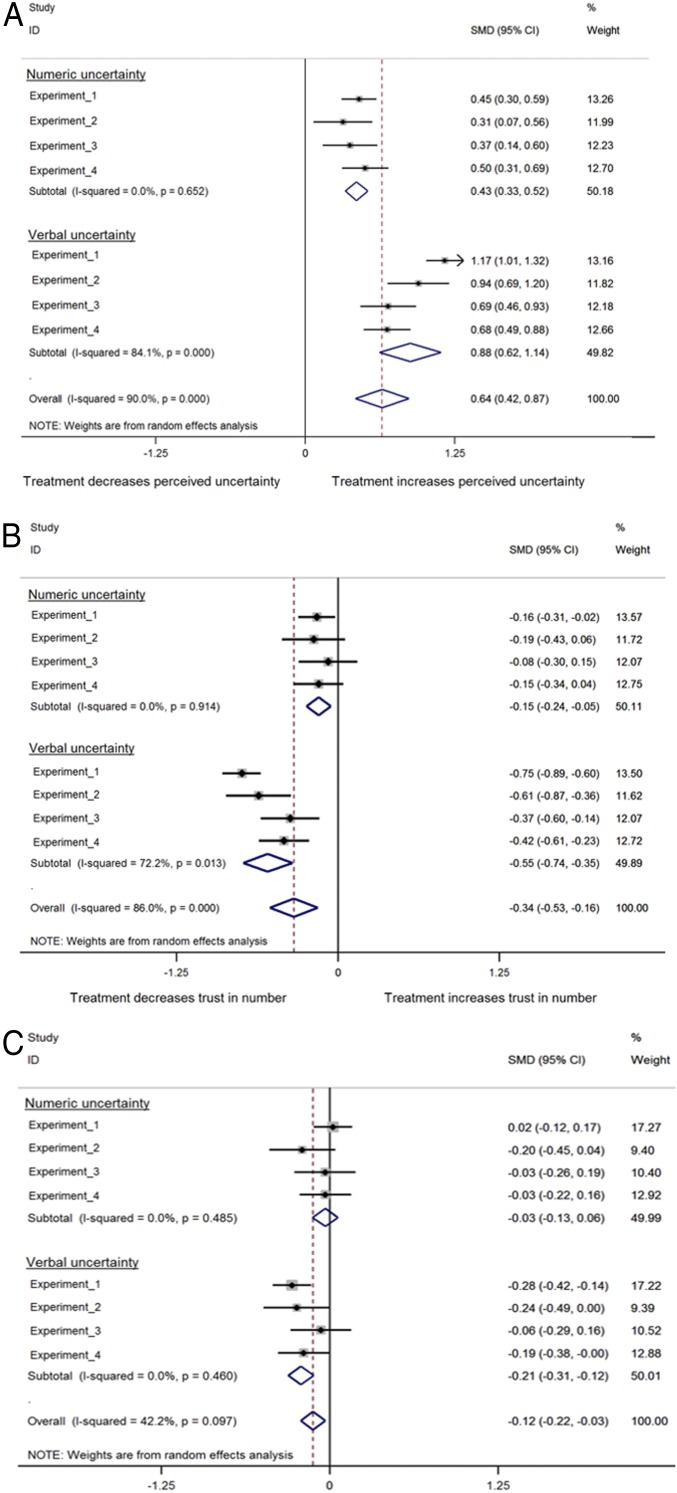
Random-effects metaanalysis. Perceived uncertainty (*A*), trust in numbers (*B*), and trust in the source (*C*).

To consolidate all of our main findings and to shed further light on the psychological effects of communicating uncertainty, we conducted a random-effects metaanalysis across all four studies for each of our key dependent variables. To ensure that the results were comparable, we only included the formats that were consistently tested across all four experiments. For ease of interpretation, we contrast “no uncertainty” (control condition) vs. “uncertainty” communication, differentiating only between “verbal” (explicit verbal statement) vs. “numeric” (numeric range with point estimate) uncertainty communication as separate subgroups. Results are presented in Fig. 5.

Overall, the communication of uncertainty in itself had a large effect on perceived uncertainty (*d* = 0.65; 95% CI [0.42; 0.87]), with the effect of verbal uncertainty (*d* = 0.88; 95% CI [0.62; 1.14]) being over twice that of numeric uncertainty (*d* = 0.43; 95% CI [0.33; 0.52]). Importantly, the communication of uncertainty did lead to a significant overall decrease in perceived reliability of the numbers (*d* = −0.34; 95% CI [−0.16; −0.53]). Although relatively small and nonsignificant across some of the studies, the weighted effect of providing numeric uncertainty on trust in numbers was also negative and significant (*d* = −0.15; 95% CI [−0.05; −0.24]). However, much of the overall effect seems to stem from verbal uncertainty, as the negative effect of verbal uncertainty on trust in numbers was much more substantial (*d* = −0.55; 95% CI [−0.35; −0.74]). Last, although the weighted effect of the communication of uncertainty across studies did also significantly and negatively influence perceived trustworthiness of the source (*d* = −0.12; 95% CI [−0.03; −0.22]), the size of the effect is similarly small and seems to be driven by verbal uncertainty (*d* = −0.21; 95% CI [−0.12; −0.31]) rather than numeric uncertainty (*d* = −0.03; 95% CI [−0.03; 0.06]).

### Field Experiment on the *BBC News* Website.

Finally, we assessed to what extent our findings would generalize beyond the context of an online laboratory experiment to a real-world setting. We therefore engaged in a unique experiment on the live *BBC News* website to test the effects of communicating uncertainty in an online news article about the United Kingdom’s labor market statistics, which are released monthly by the Office for National Statistics.

**Fig. 6.**
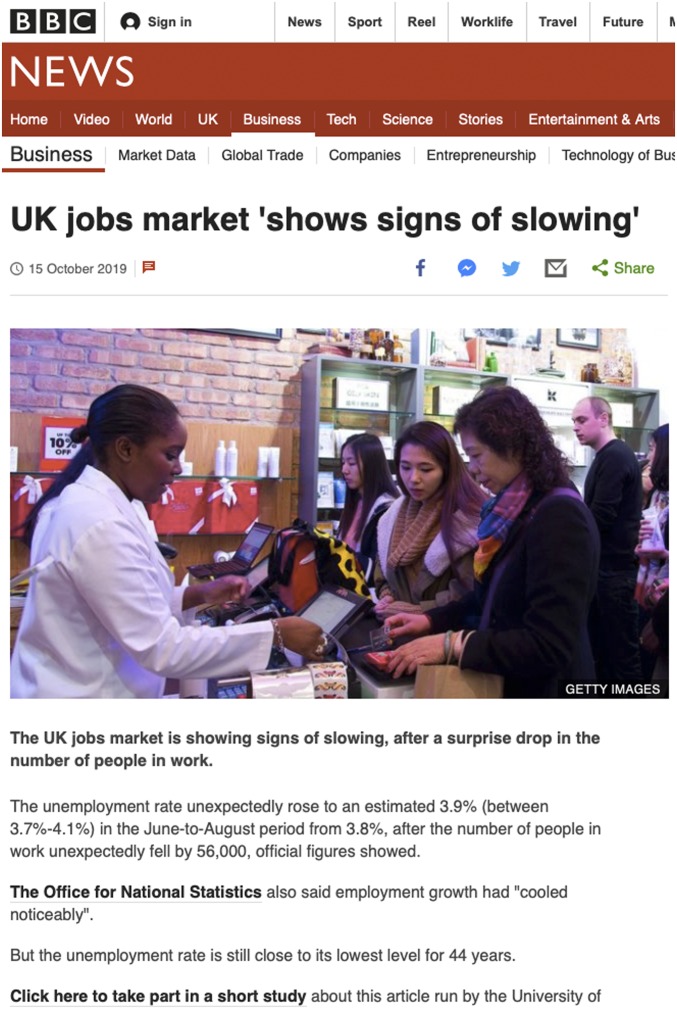
Image of the *BBC News* article that was used in experiment 5 (numerical condition: including a numeric range). Reprinted with permission from *BBC News*.

**Fig. 7.**
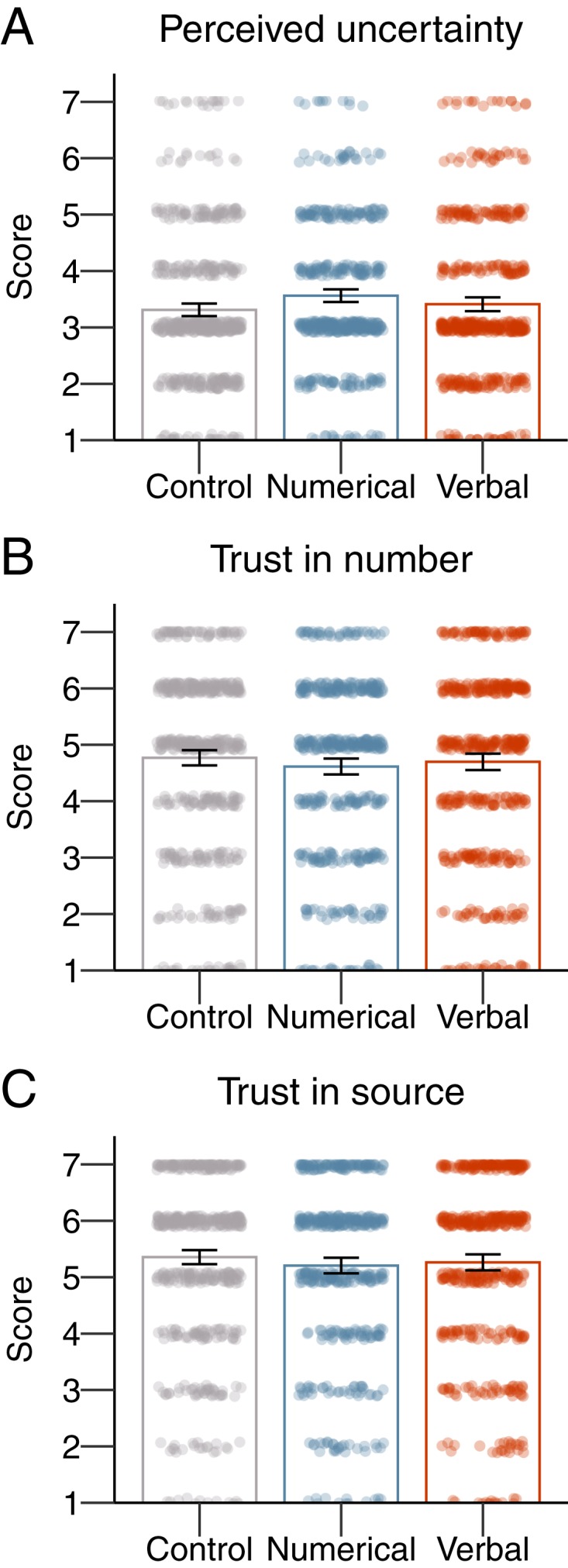
The results of field experiment 5: Means per condition for perceived uncertainty (*A*), trust in numbers (*B*), and trust in the source (*C*). The error bars represent 95% CIs around the means, and the jitter represents the distribution of the underlying data.

After a pilot experiment using a *BBC News* article about the UK labor market in September 2019, which is reported in the *SI Appendix*, we conducted an experiment with an online news article about the UK labor market on October 15, 2019. Readers of the live *BBC News* website were randomly shown one of three versions of the news article (Fig. 6). The first figure mentioned in this article was the unemployment rate, which “… unexpectedly rose to 3.9% in the June-to-August period from 3.8%, after the number of people in work unexpectedly fell by 56,000, official figures showed.” Readers were either shown this target figure without any uncertainty mentioned, as is common in all news reporting (including the BBC); with a verbal uncertainty cue (“… rose to an estimated 3.9%”), as is sometimes used in *BBC News* reporting; or with a numeric range and verbal cue [“… rose to an estimated 3.9% (between 3.7% and 4.1%)”], which is uncommon in news reporting. All other figures mentioned in the article were reported without uncertainty. After the first paragraph of the news article, which contained the figure of interest, readers were invited to take part in a short study about this article. As our survey had to be brief, we only included our key measures: After asking participants to rate their current emotional state (affect), we asked them how certain or uncertain they thought the unemployment rate figure in the story was, how trustworthy it was, and how trustworthy and competent the statisticians responsible for producing the figure were, and how trustworthy they thought the journalist responsible for producing the article was. The results are presented in Fig. 7.

The results of this field experiment showed that, in line with the laboratory experiments, people perceived the number to be more uncertain when numerical uncertainty had been communicated, compared to no uncertainty communication in the control condition. An ANOVA showed a significant main effect of uncertainty communication on perceived uncertainty [*F*_(2,__1526)_ = 4.67, *P* = 0.01; $\eta^{2}=0.006$]. Participants who read the version of the news article with a numeric range around the unemployment rate figure perceived the figure to be more uncertain than people in the control condition (M = 3.56 vs. 3.31, M_diff_ = 0.25, 95% CI [0.06; 0.44], *d* = 0.19). Participants who read the version of the news article with the verbal cue scored in between the numerical and control conditions, not significantly different from either (M = 3.41, SD *=* 1.39). This finding suggests that participants did seem to have noticed the uncertainty that was communicated.

Uncertainty communication, however, did not affect participants’ trust in the number [*F*_(2,__1526)_ = 1.20, *P* = 0.30], nor trust in the source, in this case, the statisticians responsible for producing the figures [*F*_(2,__1525)_ = 1.24, *P* = 0.29]. These findings complement the results from our laboratory experiments, which showed that a verbal cue such as “estimated” did not seem to communicate uncertainty to people and did not affect their trust in numbers or the source (as found in experiments 3 and 4). In this field experiment, we again found communicating uncertainty as a numeric range did not affect people’s trust in the source, and it also did not affect trust in the number.

In addition, the results showed no significant effects of uncertainty communication on affect [*F*_(2,__1519)_ = 0.44, *P* = 0.65], competence of the source [*F*_(2,__1525)_ = 0.61, *P* = 0.54], and trustworthiness of the journalist [*F*_(2,__1526)_ = 0.86, *P* = 0.42]. Participants’ judgments of the competence and trustworthiness of the statisticians were highly correlated (*r* = 0.80, *P* < 0.001), and on the high end of the scale (M = 5.44, SD *=* 1.41, and M = 5.28, SD *=* 1.55, respectively, out of seven); participants’ rating of the trustworthiness of the journalist was slightly lower (M = 4.61, SD *=* 1.54). These results suggest that communicating uncertainty to the participants of this field study, did not affect their (already positive) views of the trustworthiness and competence of the people involved in producing and reporting unemployment figures.

## Discussion

Centuries of human thinking about uncertainty among many leaders, journalists, scientists, and policymakers boil down to a simple and powerful intuition: “No one likes uncertainty” (1, 6, 7, 27). It is therefore often assumed that communicating uncertainty transparently will decrease public trust in science (1, 7). In this program of research, we set out to investigate whether such claims have any empirical basis. We did this by communicating epistemic uncertainty around basic facts and numbers and by systematically varying 1) the topic, 2) the magnitude of the uncertainty, and 3) the format and context through which uncertainty was communicated. We assessed the effects of uncertainty on relevant outcome measures, including cognition and trust.

Overall, we found little evidence to suggest that communicating numerical uncertainty about measurable facts and numbers backfires or elicits psychological reactance. Across five high-powered studies and an internal metaanalysis, we show that people do recognize and perceive uncertainty when communicated around point estimates, both verbally and numerically (except when only words such as “estimated” or “about” are used to imply uncertainty). In addition, uncertainty did not seem to influence their affective reaction (*SI Appendix*), and although the provision of uncertainty in general did slightly decrease people’s trust in and perceived reliability of the numbers, this effect emerged for explicit verbal uncertainty in particular.

Our research offers an important bridge between producers of statistics, communicators, and their audiences. For example, statisticians or scientists could argue that because most numeric estimates are never completely certain, presenting uncertainty around the number offers more precise information and should therefore foster more trustworthiness, not less. However, if a general audience had not considered that there might be any uncertainty around a number in the first place (e.g., around unemployment), then from a purely normative point of view people’s reaction to uncertainty in our studies is not entirely inappropriate: By providing clear variability around estimates, it is reasonable for people to adjust their level of trust in the numbers themselves. In a similar vein, one might argue that it is difficult for people to appraise the trustworthiness of a number without having access to the methodology through which the estimate is derived. However, from a social scientific standpoint, we recognize that people are frequently exposed to numbers in the news without necessarily having access to additional information, for example, about the quality of the underlying evidence (or indirect uncertainty). So how do people actually arrive at a judgment as to what numbers are reliable and trustworthy in the face of uncertainty? Although we did not set out explicitly to investigate the mechanism by which people adjust their judgments in response to uncertainty, an exploratory mediation analysis on the nationally representative sample (experiment 4) clearly suggests that the main effect of uncertainty communication (uncertainty vs. no uncertainty) on trustworthiness is fully mediated by people’s perception of the uncertainty (see *SI Appendix* for mediation analyses). In other words, this suggests that the more uncertain people perceive the numbers to be, the less reliable and trustworthy they find them. The current results help inform theoretical predictions about how people might respond to direct uncertainty about numbers, and we encourage future research to further investigate potential mechanisms as well as how people might respond to indirect uncertainty, such as additional information about the quality of the underlying evidence.

In sum, prior research has investigated whether the provision of uncertainty can help signal transparency and honesty on behalf of the communicator, or—in contrast—whether communicating uncertainty decreases trust and signals incompetence (9, 15, 17, 36). By and large, our findings illustrate that the provision of numerical uncertainty—in particular as a numeric range—does not substantially decrease trust in either the numbers or the source of the message. Verbal quantifiers of uncertainty, however, do seem to decrease both perceived reliability of the numbers as well as the perceived trustworthiness of the source. These findings were robust across topics (both contested and noncontested), mode of communication, and magnitude of uncertainty. More generally, the strong negative effects of verbal uncertainty appear consistent with prior findings that people are averse to more ambiguous statements (27, 43). As such, we hypothesize that the communication of numerical uncertainty may offer a degree of precision that reduces people’s tendency to view the admission of uncertainty as a sign of incompetence (1, 9, 36). On the other hand, across all studies, the communication of uncertainty never significantly increased perceived trust or reliability either, which is an important finding in itself and warrants further research.

Accordingly, based on these results, we therefore recommend that the communication of uncertainty around basic facts and numbers in the media is best conveyed through numerical ranges with a point estimate. This format in particular did not seem to significantly influence (i.e., reduce) perceived trust and reliability in either the number or the source of uncertainty. In addition, we draw attention to the fact that using the word “estimate” or increasing the magnitude of the confidence interval did not seem to alter people’s perception of uncertainty, which points to the need to better contextualize the degree of uncertainty for people.

Last, it is notable that we find little evidence for the motivated cognition of uncertainty (35). For example, even around more contested topics, such as global warming and immigration, although main effects were observed for people’s prior attitude toward the issue, there was no significant interaction with the communication of uncertainty. At the very least, this suggests that motivated interpretations of uncertainty do not always occur. At the same time, we must acknowledge several limitations of our program of research.

First, we recognize that people are known to struggle with psychological uncertainty about the future (44, 45), perhaps more so than uncertainty about measurable facts and numbers, an area previously neglected, and thus the focus of the current work. The context of our research was also limited, culturally, to the United Kingdom, and more contested examples for this population (e.g., around the United Kingdom’s political exit from the European Union) may have elicited different results. Moreover, while we conceptually replicated our results across multiple studies and platforms—including a preregistered national sample—we did not investigate uncertainty around more emotionally charged topics in this study, such as uncertainty about personal health outcomes (e.g., cancer), nor manipulated contestedness as an experimental factor. Indeed, there may be other circumstances (not examined here) where a significant degree of uncertainty could elicit strong emotional reactions. Finally, we attempted to improve the ecological and external validity of our manipulations by engaging in a real-world experiment on the live *BBC News* website. Although findings corroborated what we observed in controlled laboratory settings, the BBC study necessarily relied on a somewhat skewed and self-selected sample. In addition, although we generally relied on large and diverse samples, and our main effects were sufficiently powered, we may not have had sufficient power to detect very small effects in all post hoc comparisons. Sensitivity analyses showed, however, that given the sample sizes of experiments 3 and 4 (and assuming α = 0.05 and power of 0.80), we should have been able to detect small effects in these studies (*f* = 0.101, *d* = 0.20; and *f* = 0.107, *d* = 0.21, respectively). The smallest effects of interest reported in our paper are broadly beyond those thresholds (e.g., *d* = 0.26 to 0.72).

Nonetheless, even considering all of these boundary conditions, our results help inform and challenge strongly held—and often nonempirical—assumptions across domains about how the public will react to the communication of uncertainty about basic science, facts, and numbers (1, 7). A key challenge to maintaining public trust in science is for communicators to be honest and transparent about the limitations of our current state of knowledge. The high degree of consistency in our results, across topics, magnitudes of uncertainty, and communication formats suggest that people “can handle the truth.” However, if we want to effectively convey uncertainty about pressing issues, such as rising sea levels, the number of tigers left in India, the state of the economy, or how many people turn out to presidential elections; natural scientists, statisticians, and social scientists should work together to evaluate how to best present scientific uncertainty in an open and transparent manner. As such, our findings can provide valuable guidance to scientists, communicators, practitioners, and policymakers alike, who are all united by a common interest in how to effectively communicate the truth in a so-called posttruth world.

## Materials and Methods

The survey experiments were completed in a web browser and took ∼12 min to complete. For experiments 1 to 3, we recruited participants on the platform Prolific. Prolific has been found to be similar to Amazon Mechanical Turk in terms of data quality, and better suited to recruit UK-based participants (46, 47). Participants were paid £1.20 for their participation and were not allowed to participate in more than one experiment. For experiment 4, we used Qualtrics Panels to recruit a sample that was nationally representative of the United Kingdom population in terms of gender, age, and region in which the participants lived. For experiment 5, a field study, we collaborated with *BBC News* and recruited visitors of the *BBC News* website and app. This survey took ∼2 min to complete. Ethical approval for this research was granted by the Cambridge Psychology Research Ethics Committee (experiments 1, 2, and 5) and the Department of Psychology Ethics Committee (experiments 3 and 4) of the University of Cambridge. All participants gave informed consent before participation and received detailed debriefing information afterward. *SI Appendix* includes tables with an overview of the characteristics of the participants for each experiment (*SI Appendix*, Table S1) and per condition in each experiment (*SI Appendix*, Tables S2–S6), which show that the experimental groups were balanced in terms of participants’ age, gender, education level, and numeracy.

### Experiment 1.

#### Sample and design.

In experiment 1, we used a between-subjects design to test three forms of uncertainty communication (numeric vs. verbal vs. control) about three topics (tigers vs. unemployment vs. climate change). Based on a priori power calculations, which indicated we would need 1,075 people for 90% power to detect a small (interaction) effect (*f* = 0.12) when α was set at 0.05, we decided to recruit 1,125 participants (125 per cell of the design; we did this for experiment 2 and 3 as well). Three of these participants indicated to be below 18 y of age and were excluded from further analyses. The sample thus consisted of *n* = 1,122 people [769 women (68.5%); average age, 37.72; SD, 12.12; range, 18 to 72]. Compared to the UK population, this sample was relatively highly educated. Organisation for Economic Co-operation and Development data show that 18.8% of the 24- to 65-y-olds in the United Kingdom attained primary and middle school education, 35.4% upper secondary education (General Certificate of Secondary Education [GCSE] and A-levels), and 45.7% attainted tertiary education (bachelor’s, master’s, PhD, etc.) (48). In our sample, 1.6% indicated to have no educational qualifications, 38.5% indicated to have attained upper secondary education, and 59.6% indicated to have attained tertiary education. On average, political orientation of the sample was slightly leaning toward liberal (M = 3.49, SD = 1.42, on a scale from 1 = very liberal to 7 = very conservative).

#### Treatment and procedure.

After agreeing to participate, participants were asked several questions about their beliefs related to the three topics: about the conservation of endangered animals, about the present state of the country and economy, and about climate change (for more information on all measures, see *SI Appendix*). After this, participants were randomly allocated to be presented with one of nine texts. For example, the text about unemployment read as follows:

Recently, an official report came out with new information about the unemployment rate in the United Kingdom. This report stated that between April and June 2017, government statistics showed that an estimated 1,484,000 people in the UK were unemployed.

Participants in the control condition only read about this central estimate, without any information about uncertainty. For participants in the numeric uncertainty communication condition, the exact same sentence finished with a numeric range: “…unemployed (minimum 1,413,000 to maximum 1,555,000).” For participants in the verbal uncertainty communication condition, an extra sentence was added to the text: “The report states that there is some uncertainty around this estimate, it could be somewhat higher or lower.” The control text about tigers reported that “an official report stated that in 2015 an estimated 2,226 tigers were left in India.” In the numeric uncertainty communication condition, a range of minimum 1,945 to maximum 2,491 was added, and in the verbal uncertainty communication condition, the exact same sentence was used as in the unemployment condition. The text about climate change reported that “an official report stated that between 1880 and 2012, the earth’s average global surface temperature has increased by an estimated 0.85°C.” In the numeric uncertainty condition, a range of minimum 0.65 to maximum 1.06 was added, and once again the exact same sentence was used in the verbal condition (somewhat higher or lower). These numbers are based on reports by the UK Office for National Statistics (49), the International Union for Conservation of Nature Red list (50), and the Intergovernmental Panel on Climate Change (51), respectively.

#### Measures.

After reading the text, participants first reported how the information made them feel on a standard feeling thermometer from 0 = negative/unhappy to 10 = positive/happy (results are reported in the *SI Appendix*). They were then asked to recall what number was reported in the text (open question), and whether they remembered any uncertainty being implied around this number (yes, no, don’t know, don’t remember). These questions served as manipulation checks and to increase the salience of the target number. The open text responses showed that most participants were able to either correctly recall the target number (in experiment 2, where we coded all responses: 54.4%), or give a sensible estimate of the target number (experiment 2: 30.2%), indicating that generally participants understood what we meant by “this number” in the questions that followed.

Next, our key dependent variables were assessed: perceived uncertainty of the number (average of 2 items, “To what extent do you think that this number is certain or uncertain?”: 1 = very certain to 7 = very uncertain; “How much uncertainty do you think there is about this number?”: 10-point slider: not at all uncertain to very uncertain; *r* = 0.63), trust in the number (modeled after ref. 9; average of 2 items, “To what extent do you think this number is reliable [trustworthy]?”: 7-point scale from 1 = not at all to 7 = very reliable [trustworthy]; *r* = 0.88), and trust in the source (“To what extent do you think the writers of this report are trustworthy?”: 7-point scale from 1 = not at all to 7 = very trustworthy). In addition, we also asked people to how uncertain the number made them feel (10-point slider, 1 = not at all to 10 = very uncertain; results reported in *SI Appendix*). After these dependent variables, a series of unrelated variables were assessed (for more information, see *SI Appendix*). The experiment finished with questions about demographic information and a detailed debrief.

### Experiment 2.

#### Sample and design.

Experiment 2 followed on from experiment 1. Instead of varying the topics, however, we were interested in the effect of different magnitudes of uncertainty. This experiment therefore consisted of a control condition (no uncertainty communicated) plus a 2 (numeric vs. verbal uncertainty communication) × 3 (lower vs. original vs. higher magnitude of uncertainty) factorial design; so a total of seven conditions, to which participants were randomly allocated. Based on a priori power calculations, which indicated we would need 752 participants for 90% power to detect a small (*f* = 0.13) interaction effect between format and magnitude when α was set at 0.05, we recruited 877 participants from Prolific (∼125 per cell for the seven-cell design). The sample consisted of 582 women and 292 men (average age, 34.68; SD, 12.02; range, 18 to 80). Similar to experiment 1, this sample was relatively highly educated compared to the UK population: 1.1% indicated to have no educational qualifications, 39% indicated to have attained upper secondary education, and 59.8% indicated to have attained tertiary education. On average, political orientation of the sample was slightly liberal (M = 3.40; SD = 1.42).

#### Treatment and procedure.

All participants read the same text as in study 1 about unemployment, with either no uncertainty communicated, or uncertainty communicated numerically or verbally. The different magnitudes were either the original magnitude that was communicated in experiment 1, which was a numerical range of 1,413,000 to 1,555,000 (95% CI around the point estimate of 1,484,000 unemployed people) or the sentence stating that the number could be “somewhat higher or lower.” However, in addition, lower uncertainty was communicated as a range of minimum 1,448,500 to maximum 1,519,500 (a 68% CI around the point estimate, which is a range that is half as wide as the original) or through the wording “slightly higher or lower” (verbal condition). Higher uncertainty was communicated as a range of minimum 1,342,000 to maximum 1,626,000 (99.99% CI, which is a range that is twice as wide as the original) or through the wording “a lot higher or lower.” Before reading this text, participants were asked some questions about their beliefs about the state of the country and economy, and afterward, they were asked the same exact questions as in study 1 (*SI Appendix*).

### Experiment 3.

#### Sample and design.

In experiment 3, we aimed to test various other numeric and verbal uncertainty communication formats, still in the context of unemployment for consistency using a relatively high magnitude of uncertainty. This study had eight conditions, and we recruited *n* = 1,200 participants from Prolific, based on power calculations that indicated this would give us 90% power to detect a small (*f* = 0.125) effect when α was set at 0.05. The sample consisted of 806 women and 388 men (average age, 36.65; SD, 11.98; range, 18 to 85). Just as in experiments 1 and 2, the sample was relatively highly educated compared to the UK population (no educational qualifications, 1.3%; upper secondary, 34.7%; tertiary education, 63.7%) and on average slightly leaning toward a liberal political orientation (M = 3.40; SD = 1.38). After answering questions about their beliefs about the state of the country and economy, people read a version of the following text, which was designed to read more like a short news article to increase ecological validity:

**UK unemployment drops**

Official figures from the first quarter of 2018 show that UK unemployment fell by 116,000 compared with the same period last year.

This puts the total number of people who are unemployed at 1.42 million.

The number of those in work increased and wage growth improved over the same period. However, weak incomes have been a problem for a decade. “It will take a long period of wages rising above the rate of inflation for people to feel significantly better off,” one economics commentator is quoted as saying.

This version served as the control condition. We tested three numeric uncertainty communication formats, three verbal formats, and a mixed numeric/verbal format (Table 1).

#### Measures.

After reading the text, participants were asked the same questions as in experiments 1 and 2, except now specified for each number (the fall in unemployment and the total number of unemployed people) for the recall question, their perception of uncertainty around the numbers (α = 0.80) and trust in the numbers (α = 0.92). For the analyses, answers were averaged across both numbers given that there were no meaningful or significant differences between the two. Trust in the source was assessed with the item “To what extent do you think the civil servants who are responsible for these unemployment figures are trustworthy?” on a scale from 1 = not at all to 7 = very trustworthy. In addition, we asked people to what extent they thought journalists who write articles such as the one they read were trustworthy, and to what extent they thought government statistics in general were reliable (on scales from 1 = not at all to 7 = very trustworthy [reliable]).

### Experiment 4.

#### Sample and design.

Experiment 4 was preregistered at aspredicted.org (https://aspredicted.org/blind.php?x=d3xu67). We recruited 1,050 adults who lived in the United Kingdom to participate in this study via Qualtrics Panels, based on power calculations that indicated we would need 995 participants to have 90% power to detect a small (*f* = 0.125) effect when α was set at 0.05. This sample was nationally representative of the general UK population in terms of age, gender, and geography quotas (51% female; mean age, 45.34 y; SD, 16.47; age range, 18 to 86). In this sample, 8.9% of the participants had no educational qualifications, 44.8% had attained upper secondary education, and 46.1% had tertiary education. On average, the sample was again slightly leaning liberal (M = 3.74; SD = 1.51).

#### Treatment and procedure.

We aimed to test whether we would find the same results when communicating uncertainty around a more contentious topic, so we presented people with a text about migration statistics based on a *BBC News* article of these Office of National Statistics figures (52):

**Migration figures: EU migration still adding to UK population**

Official figures from last year show that there were 101,000 more people coming to the UK from the EU than leaving in 2017. This is the lowest EU net migration figure since 2013, but it means that EU migrants are still adding to the UK population.

Net migration is the difference between the number of people coming to live in the UK for at least 12 months and those emigrating. The 2017 overall net migration figure (both from the EU and non-EU countries) is also down, from record highs in 2015 and early 2016.

However, “The figures show that the government remains a long way off from meeting its objective to cut overall net migration, EU and non-EU, to the tens of thousands,” one Home Affairs correspondent is quoted as saying.

This experiment had five conditions (Table 1): Besides the control condition (above, no uncertainty), uncertainty was communicated numerically with a range after the point estimate, or via “+/− two standard errors”; or verbally using the word “around” before the estimate of 101,000, or with an explicit verbal statement.

#### Measures.

Before reading the text, participants answered demographic questions and questions about their beliefs about the state of society and the economy and their attitudes toward migration [with three items from the European Social Survey (53): “Would you say it is generally good for the UK’s economy that people come to live here from other countries?”: 1 = very bad for the economy to 7 = very good for the economy; “Would you say that the UK’s cultural life is generally undermined or enriched by people coming to live here from other countries?”: 1 = cultural life undermined to 7 = cultural life enriched; “Is the UK made a worse or a better place to live by people coming to live here from other countries?”: 1 = worse place to live to 7 = better place to live; α = 0.91]. After reading the text, participants answered the same questions as in experiments 1 and 2. Perceived trustworthiness of the source was assessed with the item, “To what extent do you think the civil servants who are responsible for these migration figures are trustworthy?” on a scale from 1 = not at all to 7 = very trustworthy. In addition, we asked people to what extent they thought the conclusions based on the number; the news article they just read; and journalists who write articles such as the one they read were trustworthy, and to what extent government statistics in general were reliable (on scales from 1 = not at all to 7 = very trustworthy [reliable]).

### Experiment 5: Field Experiment with *BBC News*.

#### Sample and design.

For this field experiment, we worked with *BBC News* and the BBC’s Head of Statistics. After gaining experience with the process of running a field experiment in this context during a Pilot Study in September 2019 (*SI Appendix*), we conducted the experiment on October 15, 2019, using *BBC News Online*’s coverage of the October Labor Market Release from the UK Office for National Statistics. After the labor market figures were released, we worked with the relevant journalists and the Head of Statistics to select a target figure to communicate uncertainty before the news article was published on the website. The journalists were responsible for the content of the news article. The target figure we selected was the UK unemployment rate, which was the first figure mentioned in the news story (Fig. 6). The field experiment had three conditions: visitors of the website were randomly shown a version of the news article in which the target figure was presented without any uncertainty (“… rose to 3.9%”); with a verbal uncertainty cue (“… rose to an estimated 3.9%”); or with a numeric range and verbal cue [“… rose to an estimated 3.9% (between 3.7 and 4.1%)”]. At the bottom of the first paragraph of the news article, readers were invited to “Click here to take part in a short study about this article run by the University of Cambridge.”

*BBC News* website visitors were able to participate in the study for about 24 h. During that time, 2,462 people clicked on the survey link, which took people to the starting page of the online survey with information about the study and informed consent. The survey was completed by 1,700 people (18 of whom completed the dependent variables but not demographics): 549 people in the control condition, 557 in the numeric condition, and 594 in the verbal condition. A technical issue that was created when the journalistic team updated the story after its first release resulted in participants in both experimental conditions also being shown the control condition version of the story, without any uncertainty mentioned, between 10:00 AM and 10:49 AM UK time. We therefore had to exclude all participants in the experimental conditions who participated between in that time frame, which were 69 participants in the numerical and 94 in the verbal condition. We also excluded five participants who reported to be below 18 y of age, and one outlier who reported being 114 y old (which was extremely unlikely). The final sample consisted of 1,531 people: 520 participants in the control condition, 463 in the numerical condition, and 470 in the verbal condition. We had no control over the exact number of people that would participate in this field study, so we conducted a sensitivity analysis to compute the effect size that we should be able to detect with 1,531 participants in three groups, α = 0.05 and 90% power: which is a small effect, *f* = 0.09. There were 1,131 men (73.9%) and 344 women (22.5%) who participated, with an average age of 44.82 (SD, 15.29; range, 18 to 86). The sample was relatively highly educated: 30.5% indicated to have obtained a higher degree (MSc, PhD, or equivalent), 43.6% a bachelor’s degree, 22.5% school (GCSE, A-level, or equivalent), and 1% indicated to have not completed formal education.

#### Measures.

After reading information about the study and providing informed consent, participants first answered a question about their current affective state, “How does the information you just read make you feel?”: on a feeling thermometer from 0 = negative/unhappy to 10 = positive/happy, and subsequently a comprehension question (*SI Appendix*). Next, we assessed perceived uncertainty (“How certain or uncertain do you think the unemployment rate figure in the story is?”: on a scale from 1 = very certain to 7 = very uncertain), trust in the number (“How trustworthy do you think the unemployment rate figure in the story is?”: 1 = not at all trustworthy to 7 = very trustworthy), trust in the source (“How trustworthy do you think the statisticians responsible for producing the figure are?”: 1 = not at all trustworthy to 7 = very trustworthy), competence of the source (“How competent do you think the statisticians responsible for producing the figure are?”: 1 = not at all competent to 7 = very competent), and trust in the journalistic source (“How trustworthy do you think the journalist responsible for writing the story is?”: 1 = not at all trustworthy to 7 = very trustworthy). The questionnaire finished with asking participants for their age, gender, and the highest level of education they had completed.

### Data Availability.

The datasets collected and analyzed in the reported studies are available on the Open Science Framework, https://osf.io/mt6s7/ (DOI:10.17605/OSF.IO/MT6S7).

## Supplementary Material

Supplementary File

## Footnotes

*Effects on people’s emotions were of secondary interest and are reported in detail in the *SI Appendix*.
