# Supplementary material for: The effects of communicating uncertainty on public trust in facts and numbers
Source: Proc Natl Acad Sci U S A. 2020 Mar 23;117(14):7672–83. doi: 10.1073/pnas.1913678117 (PMC7149229; doi:10.1073/pnas.1913678117)
Supplement: Supplementary File [file pnas.1913678117.sapp.pdf]

Supplementary Information for

The effects of communicating uncertainty on public trust in facts and numbers

Correspondence to:

a.m.van.der.bles@rug.nl or sander.vanderlinden@psychol.cam.ac.uk

Table of Content

Overview of participant characteristics for and within each Experiment 2
Experiment 1 8
  Additional Methodological Information... 8
  Additional Results 10
Experiment 2 15
  Additional Methodological Information... 15
  Additional Results 16
Experiment 3 19
  Additional Results 20
Experiment 4 21
  Additional Results 21
  Mediation Analyses 24
Experiment 5: Field experiment.. 27
  Pilot study.. 27
  Additional Results 28
References 29

www.pnas.org/cgi/doi/10.1073/pnas.1913678117

Overview of participant characteristics for and within each experiment

Table S1. Overview of demographic information about the participants of each experiment.

Experiment	Recruitment	*N*	% women	Age		% higher education	Numeracy *M* (*SD*)
				M (*SD*)	range		
1	Prolific	1122	68.5%	37.72 (12.12)	18-72	59.6%	2.14 (1.11)
2	Prolific	877	66.4%	34.68 (12.02)	18-80	59.8%	2.27 (1.16)
3	Prolific	1200	67.2%	36.65 (11.98)	18-85	63.7%	2.22 (1.11)
4	Qualtrics Panels	1050	51%	45.34 (16.47)	18-86	46.1%	1.78 (0.98)
5	BBC News website	1531	22.5%	44.82 (15.29)	18-86	74.1%	

Note. *N* = sample size; % women = percentage women in sample; age *M (SD)* = mean age and standard deviation; % higher education = percentage of people who indicated they had attained tertiary education (a Bachelors, Masters, or doctoral degree, or equivalent); Numeracy scores ranged from 1 to 4, *M (SD)* = mean numeracy and standard deviation. Numeracy was not assessed in Experiment 5. To compare the education level in our samples to the national population in the UK: recent OECD data about education level in the UK shows that 45.7% of the 24-65-year-olds in the UK attainted tertiary education (Bachelors, Masters, PhD, etc.); 18.8% attained primary and middle school education, and 35.4% upper secondary education (GSCE & A-levels).

Table S2. Overview of participants' gender, age, education level, and average numeracy per condition of the design of Experiment 1.

	Uncertainty Communication Format		
Topic	Control (no uncertainty)	Numerical	Verbal
Tigers			
n	121	126	126
% Women	67.8%	61.1%	69.8%
Mean age (SD)	38.27 (12.89)	37.34 (12.40)	37.23 (11.73)
% Higher education	51.2%	49.2%	50.8%
Mean numeracy (SD)	2.26 (1.14)	2.10 (1.07)	2.19 (1.15)
Climate science			
n	124	124	126
% Women	73.4%	65.3%	69%
Mean age (SD)	37.24 (11.71)	36.54 (11.98)	36.02 (11.71)
% Higher education	41.1%	48.4%	52.4%
Mean numeracy (SD)	2.11 (1.19)	2.15 (1.15)	2.23 (1.15)
Unemployment			
n	128	123	124
% Women	68%	74%	68.5%
Mean age (SD)	39.46 (11.98)	39.52 (12.78)	37.90 (11.80)
% Higher education	49.2%	55.3%	49.2%
Mean numeracy (SD)	2.12 (1.06)	1.98 (1.04)	2.16 (1.06)
Total across topics			
n	373	373	376
% Women	69.7%	66.8%	69.1%
Mean age (SD)	38.34 (12.20)	37.80 (12.42)	37.04 (11.74)
% Higher education	47.2%	50.9%	50.8%
Mean numeracy (SD)	2.16 (1.13)	2.08 (1.09)	2.19 (1.12)

Note. Participants were recruited via Prolific Academic. *n* = size of subsample; % women = percentage women in subsample; age *M (SD)* = mean age and standard deviation in subsample; % higher education = percentage of people in the subsample who indicated they had obtained a Bachelors, Masters, or doctoral degree, or equivalent; Numeracy *M (SD)* = mean numeracy and standard deviation in subsample.

Table S3. Overview of participants' gender, age, education level, and average numeracy per condition of the design of Experiment 2.

	Uncertainty Communication Format		
Magnitude	Control (no uncertainty)	Numerical	Verbal
Actual uncertainty (replication Exp.1)			
n	130	126	126
% Women	64.6%	64.3%	68.3%
Mean age (SD)	34.42 (12.69)	35.02 (12.14)	35.31 (12.96)
% Higher education	43.8%	55.6%	50%
Mean numeracy (SD)	2.28 (1.17)	2.37 (1.20)	2.41 (1.20)
Higher magnitude of uncertainty			
n		126	117
% Women		59.5%	65.8%
Mean age (SD)		33.67 (11.18)	34.72 (11.31)
% Higher education		50%	43.6%
Mean numeracy (SD)		2.39 (1.12)	2.32 (1.22)
Lower magnitude of uncertainty			
n		126	126
% Women		72.2%	69.8%
Mean age (SD)		34.82 (12.24)	34.79 (11.66)
% Higher education		47.6%	55.6%
Mean numeracy (SD)		2.16 (1.13)	2.01 (1.05)

Note. Participants were recruited via Prolific Academic. *n* = size of subsample; % women = percentage women in subsample; age *M (SD)* = mean age and standard deviation in subsample; % higher education = percentage of people in the subsample who indicated they had obtained a Bachelors, Masters, or doctoral degree, or equivalent; Numeracy *M (SD)* = mean numeracy and standard deviation in subsample.

Table S4. Overview of participants' gender, age, education level, and average numeracy per condition of the design of Experiment 3.

Uncertainty Communication Format	*n*	% women	Age *M* (*SD*)	% higher education	Numeracy *M* (*SD*)
Control – no uncertainty	152	64.5%	36.83 (11.35)	50.7%	2.32 (1.13)
Numerical +/-	147	73.5%	34.65 (10.77)	49%	2.20 (1.13)
Numerical range (with point estimate)	145	59.3%	36.50 (12.11)	49%	2.15 (1.10)
Numerical range (without point estimate)	148	66.9%	38.21 (12.11)	50.7%	2.16 (1.11)
Verbal cue "estimates"	150	68%	37.61 (13.55)	52%	2.34 (1.09)
Verbal explicit uncertainty statement	149	70.5%	36.20 (11.98)	51.7%	2.13 (1.06)
Verbal implicit uncertainty statement	154	67.5%	35.95 (11.56)	55.2%	2.34 (1.16)
Mixed Numerical/Verbal	155	67.1%	37.17 (12.17)	49.7%	2.09 (1.10)

Note. Participants were recruited via Prolific Academic. *n* = size of subsample; % women = percentage women in subsample; age *M (SD)* = mean age and standard deviation in subsample; % higher education = percentage of people in the subsample who indicated they had obtained a Bachelors, Masters, or doctoral degree, or equivalent; Numeracy *M (SD)* = mean numeracy and standard deviation in subsample.

Table S5. Overview of participants' gender, age, education level, and average numeracy per condition of the design of Experiment 4.

Uncertainty Communication Format	*n*	% women	Age *M* (*SD*)	% higher education	Numeracy *M* (*SD*)
Control – no uncertainty	210	51.4%	45.82 (15.96)	35.2%	1.74 (0.96)
Numerical +/-	210	53.3%	44.35 (16.61)	42.9%	1.83 (1.01)
Numerical range (with point estimate)	210	46.2%	46.89 (16.09)	38.6%	1.81 (0.91)
Verbal cue "around"	210	52.4%	43.65 (16.54)	33.3%	1.69 (0.98)
Verbal explicit uncertainty statement	210	51.4%	45.98 (17.07)	34.8%	1.82 (1.05)

Note. Participants were recruited by Qualtrics Panels. *n* = size of subsample; % women = percentage women in subsample; age *M (SD)* = mean age and standard deviation in subsample; % higher education = percentage of people in the subsample who indicated they had obtained a Bachelors, Masters, or doctoral degree, or equivalent; Numeracy *M (SD)* = mean numeracy and standard deviation in subsample.

Table S6. Overview of participants' gender, age, education level, and average numeracy per condition of the design of field Experiment 5.

Uncertainty Communication Format	*n*	% women	Age *M* (*SD*)	% higher education
Control – no uncertainty	548	24.1%	45.01 (15.62)	72.6%
Verbal cue "estimated"	489	22.7%	44.69 (14.97)	76.3%
Numerical range (and verbal cue)	485	20.4%	44.75 (15.29)	73.4%

Note. Participants were recruited by Qualtrics Panels. *n* = size of subsample; % women = percentage women in subsample; age *M (SD)* = mean age and standard deviation in subsample; % higher education = percentage of people in the subsample who indicated they had obtained a Bachelors, Masters, or doctoral degree, or equivalent; Numeracy was not assessed in this field experiment.

Experiment 1

Table S7. Overview of the experimental conditions and treatment texts of Experiment 1.

	Uncertainty Communication Format		
**Topic**	**Control (no uncertainty)**	**Numerical**	**Verbal**
**Tigers**	*Recently, an official report came out with new information about the number of tigers in India. This report stated that in 2015, there were an estimated 2,226 tigers left in India.*	*…an estimated 2,226 tigers left in India (minimum 1,945 to maximum 2,491).*	*…an estimated 2,226 tigers left in India. The report states that there is some uncertainty around this estimate, it could be somewhat higher or lower.*
**Climate science**	*Recently, an official report came out with new information about global warming. This report stated that between 1880 and 2012, the earth's average global surface temperature has increased by an estimated 0.85ºC.*	*…an estimated 0.85ºC (minimum 0.65 to maximum 1.06).*	*…an estimated 0.85ºC. The report states that there is some uncertainty around this estimate, it could be somewhat higher or lower.*
**Unemployment**	*Recently, an official report came out with new information about the unemployment rate in the United Kingdom. This report stated that between April and June 2017, government statistics showed that an estimated 1,484,000 people in the UK were unemployed.*	*…were unemployed (minimum 1,413,000 to maximum 1,555,000).*	*…were unemployed. The report states that there is some uncertainty around this estimate, it could be somewhat higher or lower.*

Additional Methodological Information

Prior beliefs. In order to be able to test for motivated cognition effects, we assessed people's beliefs related to the topics we used in our manipulation (tigers in India, climate science, unemployment) prior to participants' reading the manipulation texts. These questions were presented to the participants in the same order as presented here. We assessed *beliefs about the conservation of endangered animals* with three items that were designed for this

study (“Do you believe that it is important for humans to protect endangered animals from becoming extinct?” and “Do you believe that it is important for humans to promote the conservation of endangered animals?” on a scale from 1 = *not important at all* to 7 = *very important*, and “Do you think people should be doing more or less to promote the conservation of endangered animals?” on a 7 scale from 1 = *much less* to 7 = *much more*; α = .90).

Belief about unemployment was measured with one item (“What do you think overall about the state of unemployment in the United Kingdom nowadays?” on a scale from 1 = *very bad, a lot of unemployment* to 7 = *very good, little unemployment*; designed for this study), which was measured amongst items assessing *satisfaction with the (economic) state of the country* (two items(1): “On the whole, to what extent are you dissatisfied or satisfied with the way things are going in the United Kingdom today?” and “On the whole, how satisfied are you with the present state of the economy in the United Kingdom?”, 1 = *very dissatisfied* to 7 = *very satisfied*, r = .72). In addition, we assessed *Zeitgeist of societal discontent*, which was measured on a scale with 7 items (adapted from Van der Bles et al.(2); “To what extent does the average person in the United Kingdom suffer from, or is bothered by… crime; financial problems; discrimination; indecent or antisocial behavior by others; injustice; corruption or fraud; immigration; on a scale from 1 = *not at all* to 7 = *a great deal*, α = .86).

Climate change beliefs were measured with four items(3) (α = .89): “How strongly do you believe that global warming is or is not happening?” (scale from 1 = *I strongly believe global warming is NOT happening*, via 4 = *I am unsure whether or not global warming is happening*, to 7 = *I strongly believe global warming IS happening*); “Do you believe that global warming is caused by natural changes in the environment or by human activities?” (scale from 1 = *mostly by natural changes in the environment*, via 4 = *equally by natural changes and human activities,* to 7 = *mostly by human activities*); “How worried are you about global warming?” (scale from 1 = *not worried at all,* via 4 = *moderately worried,* to 7 = *very worried*); and “Do you think people should be doing more or less to reduce global warming?” (scale from 1 = *much less*, via 4 = *same amount,* to 7 = *much more*).

Additional variables. After measuring these prior beliefs, we presented participants with one of 9 manipulation texts, and subsequently asked them to indicate their current affective state on a feeling thermometer, to recall the number they had just read and whether there was any uncertainty presented around it; after which we assessed our key dependent variables (as described in the Method in the main text). After this, we included a series of

variables in our experiment for exploratory purposes that fall outside of the scope of this paper. Among these variables, we assessed participants' *mood* with 5 positive (α = .81, e.g. "inspired") and 5 negative (α = .87, e.g. "upset") items (4), scale from 1 = *very slightly or not at all,* to 5 = e*xtremely*), the results of which are reported below in the Additional Results section. We also included several additional variables for exploratory purposes in the survey that fall outside the scope of this paper and thus not further reported here.

Demographic variables. At the end of the survey, we asked participants about their age, gender, nationality, education level, vote in the Brexit referendum, and political orientation ("Here is a 7-point scale on which the political views that people might hold are arranged from very liberal to very conservative. Where would you place yourself on this scale?" from 1 = *very liberal* to 7 = *very conservative*).

Additional Results

In this first experiment, 1122 participants read a short text about one of three topics (tigers in India, climate science, or unemployment), that contained either no uncertainty (control condition), uncertainty communicated as a numerical range, or uncertainty communicated as a verbal statement. The main text reports results collapsed across all three topics, focusing on the main effect of uncertainty communication in numerical and verbal format. Here, we present analyses in which we examine both the effect of the format of uncertainty communication as well as the effect of the context or topic that the number is about, and their potential interaction. Results are reported for the key dependent variables that are also described in the main text, as well as for affective responses and the extent to which people reported the numbers made them feel uncertain.

Perceived uncertainty of the number. An analysis of variance (ANOVA) testing the effect of topic and uncertainty communication format on perceived uncertainty of the number revealed no significant main effect of topic, but a significant main effect of communication format, $F(2, 1113) = 139.92$, $p < .001$; $\eta_p^2 = .20$, and a small significant interaction effect, $F(4, 1113) = 2.78$; $p = .026$; $\eta_p^2 = .01$. Figure S1 presents the results. As reported in the main text, participants perceived the number to be significantly more uncertain when uncertainty was communicated to them in a verbal format, as compared to numerical or no uncertainty. Inspecting means per condition revealed that the interaction effect was driven by how people responded to uncertainty as a numerical range around the climate science estimate. Whereas participants perceived the unemployment and tiger numbers to be significantly more uncertain

when uncertainty was communicated numerically as compared to control, and again when it was communicated verbally (see Figure S1 panel A), participants who read about climate science did not perceive significantly more uncertainty around this number when uncertainty was communicated numerically compared to control (pairwise comparisons: M = 4.42 vs. 4.11, M_{diff} = 0.31, 95%CI[-0.04;0.66], d = 0.23). Participants did perceive the climate science number to be significantly more uncertain when it was communicated verbally compared to control (pairwise comparisons: M = 5.86 vs. 4.11, M_{diff} = 1.75, 95%CI[1.40;2.09], d = 1.25).

Trust in the number. We also asked people to indicate how reliable and trustworthy they thought the numbers were. A two-way ANOVA revealed a main effect of topic ($F(2, 1113) = 28.32$, $p < .001$; $\eta_p^2 = .05$) and a main effect of uncertainty communication format ($F(2, 1113) = 64.73$, $p < .001$; $\eta_p^2 = .10$), but no interaction. Figure S1 (panel B) presents the results. Post-hoc comparisons (Tukey HSD) showed that across topics, people who were presented with verbal uncertainty communication trusted the numbers less than people in the control condition (M = 3.51 vs. 4.52, M_{diff} = -1.01, 95%CI[-1.23;-0.79], d = 0.75). We found no significant difference between trust in numbers between the numerical uncertainty and control conditions (M = 4.31 vs. 4.52, M_{diff} = -0.21, 95%CI[-0.43;0.01], d = 0.17). Across topics, people reported less trust in unemployment numbers than the number of tigers (M = 3.72 vs. 4.39, M_{diff} = -0.67, 95%CI[-0.89;-0.45], d = 0.49) or climate science estimates (M = 3.72 vs. 4.22, M_{diff} = -0.51, 95%CI[-0.73;-0.29], d = 0.36). The lack of an interaction effect means that uncertainty communication did not have a different impact on people's trust in numbers about different topics.

Trust in the source. In addition, we asked people how trustworthy they thought "the writers of the report" were. A two-way ANOVA again showed a main effect of uncertainty communication format ($F(2, 1113) = 11.66$, $p < .001$; $\eta_p^2 = .02$) and of topic ($F(2, 1113) = 24.56$, $p < .001$; $\eta_p^2 = .04$), and no interaction. Panel C of Figure S1 presents the results. As was reported in the main text, verbal uncertainty communication across all topics significantly reduced trust in the source compared to no uncertainty communication, but numerical uncertainty communication did not. Across all uncertainty communication formats, trust in the source ("writers of the report") was higher for people who read about tigers in India compared to climate science (M = 4.76 vs. 4.45, M_{diff} = 0.31, 95%CI[0.09;0.52], d = 0.25). People who read about unemployment reported significantly less trust in the source compared to both people who read about tigers (M = 4.13 vs. 4.76, M_{diff} = -0.63, 95%CI[-0.84;-0.42], d = 0.51) and climate change (M = 4.13 vs. 4.45, M_{diff} = 0.32, 95%CI[-0.54;-0.11], d = 0.25).

Feeling uncertain. In addition to these key dependent variables, we also asked people to what extent the number made them feel uncertain. Results of a two-way ANOVA showed a significant main effect of communication format ($F(2, 1113) = 44.91$, $p < .001$; $\eta_p^2 = .08$) and no significantly main effect of topic nor an interaction. Tukey's HSD post-hoc comparisons revealed that people reported to feel significantly more uncertain after being presented with verbal uncertainty information, as compared to numerical ($M = 6.24$ vs. 5.13, $M_{\text{diff}} = 1.11$, 95%CI[0.74;1.48], $d = 0.52$) or no uncertainty information ($M = 6.24$ vs. 4.80, $M_{\text{diff}} = 1.44$, 95%CI[1.06;1.81], $d = 0.66$).

The results of Experiment 1 thus indicate that while the context of the numbers did influence people's overall trust in the numbers and the source, it did not interact with uncertainty communication: trust in numbers and the source was not affected differently by uncertainty communication for the different topics we tested. We only found a small significant interaction effect for perceived uncertainty of the number, in which it seems that in the context of climate change people did react slightly differently to numerical uncertainty communication. We therefore decided to focus on one topic only in Experiment 2 and 3 (both unemployment) and 4 (migration).

Affective responses. Directly after the manipulation text, we asked people to indicate how the information they just read made them feel on a feeling thermometer from 1 = *negative/unhappy* to 10 = *positive/happy*. A two-way analysis of variance (ANOVA: topic x uncertainty communication format) showed a main effect of topic ($F(2, 1113) = 16.47$; $p < .001$; $\eta_p^2 = .03$), and no significant main effect of uncertainty communication nor interaction. Post-hoc paired comparisons (Tukey's HSD) showed that participants who read about the number of tigers in India, regardless of whether uncertainty was communicated, felt more negative than participants who read about climate science ($M = 2.39$ vs. 3.18, $M_{\text{diff}} = -0.79$, 95%CI[-1.16;-0.42], $d = 0.38$) or unemployment ($M = 2.39$ vs. 3.18, $M_{\text{diff}} = -0.79$, 95%CI[-1.16;-0.42], $d = 0.36$). Uncertainty communication did not lead to differences in how people reported the information made them feel.

In addition, we assessed participants mood (with the Positive and Negative Affect Scale, 5-point scale) after all key dependent measures. A two-way ANOVA of topic and uncertainty communication format showed no significant main effects nor interaction for positive mood (total $M = 2.74$, $SD = 0.84$). A two-way ANOVA for negative mood showed a small significant main effect of topic ($F(2, 1113) = 3.81$; $p = .022$; $\eta_p^2 = .007$), and no significant main effect of uncertainty communication nor interaction. Post-hoc paired

comparisons (Tukey's HSD) showed that participants who read about climate science, regardless of uncertainty being communicated or not, reported more negative mood than participants who read about unemployment (M = 1.64 vs. 1.48, M_{diff} = 0.15, 95%CI[0.02;0.28], d = 0.20). Participants who read about tigers in India did not score significantly different from either two (M = 1.55, SD = 0.73).

These results suggest that while people's affective reactions were influenced by the topic about which they read, uncertainty communication did not influence mood or affective reactions; neither directly after the manipulation, nor later in the survey. In Experiments 2, 3, and 4, we continued to include the feeling thermometer directly after the manipulation to assess immediate people's affective response, but we did not assess mood in these studies.

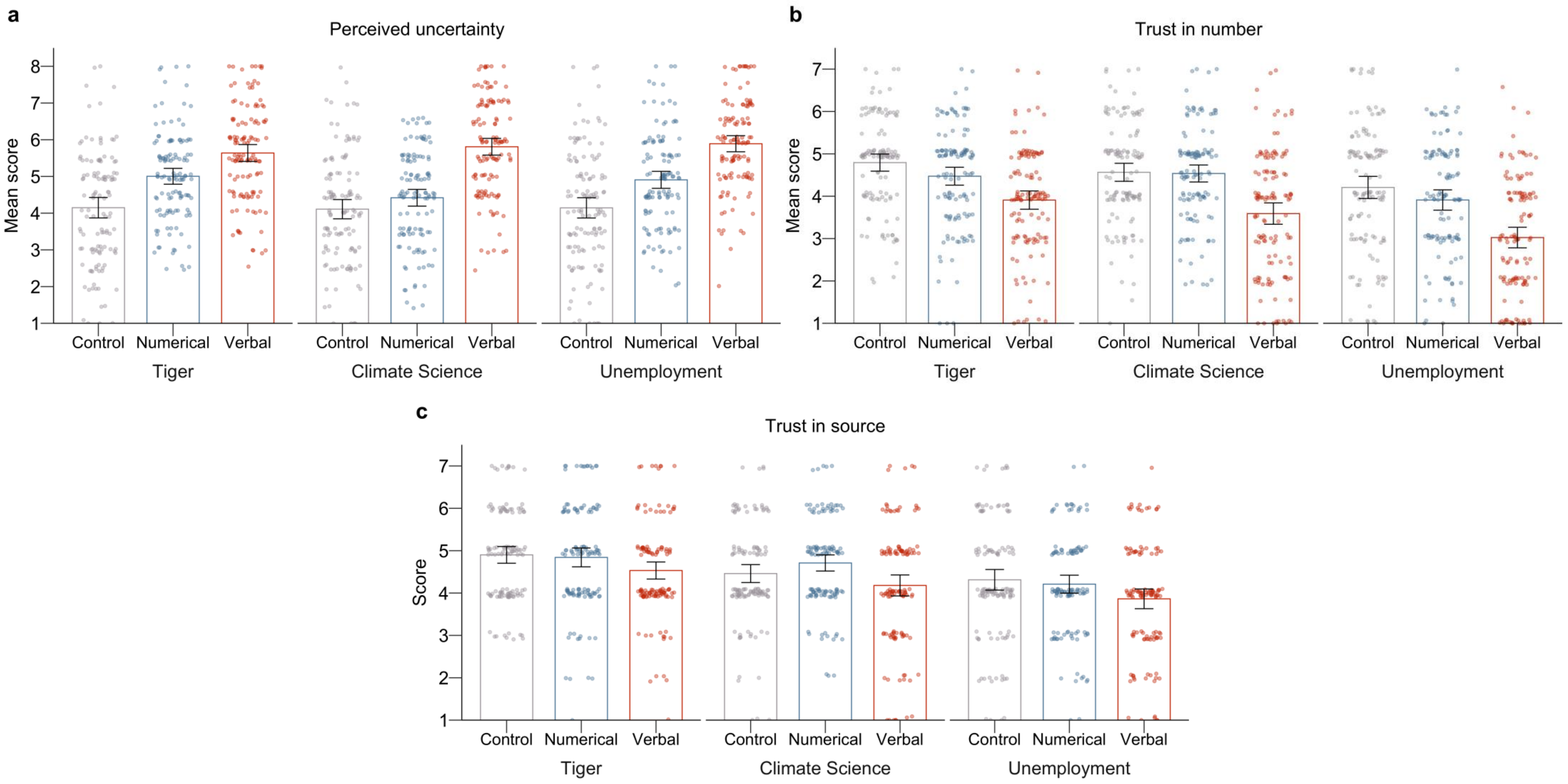

Figure S1. The results of Experiment 1: Means per condition for each topic for perceived uncertainty (panel a), trust in numbers (panel b), and trust in the source (panel c). The error bars represent 95% confidence intervals around the means and the jitter represents the distribution of the underlying data.

Experiment 2

Table S8. Overview of the experimental conditions and treatment texts of Experiment 2.

**Control condition:**	*Recently, an official report came out with new information about the unemployment rate in the United Kingdom. This report stated that between April and June 2017, government statistics showed that an estimated 1,484,000 people in the UK were unemployed.*	
	**Format**	
**Magnitude**	**Numerical**	**Verbal**
Lower uncertainty	*…were unemployed (minimum 1,448,500 to maximum 1,519,500).*	*…were unemployed. The report states that there is some uncertainty around this estimate, it could be slightly higher or lower.*
Original uncertainty	*…were unemployed (minimum 1,413,000 to maximum 1,555,000).*	*…were unemployed. The report states that there is some uncertainty around this estimate, it could be somewhat higher or lower.*
Higher uncertainty	*…were unemployed (minimum 1,342,000 to maximum 1,626,000).*	*…were unemployed. The report states that there is some uncertainty around this estimate, it could be a lot higher or lower.*

Additional Methodological Information

Participants first answered questions about their *belief about unemployment*, *satisfaction with the (economic) state of the country* (r = .71), and *Zeitgeist of societal discontent* (α = .85; same measures as Experiment 1). Subsequently, they were presented with one of seven manipulation texts, after which we asked them to indicate how they felt on a feeling thermometer, we assessed comprehension checks, and our key dependent variables, with the same materials as in Experiment 1 (reported in the main text). We added one item to our set of key dependent variables that measured *perceived reliability of government statistics* ("To what extent do you think government statistics are reliable?" on a scale from 1 = *not at*

all to 7 = *very reliable*). Just as in Experiment 1, we included additional variables for exploratory purposes in the survey that fall outside the scope of this paper and thus not further reported here. The survey again finished with the assessment of the same demographic variables.

Additional Results

Replicating Experiment 1. The control condition and "original magnitude" numerical and verbal uncertainty conditions of Experiment 2 enabled us to conduct a direct test of whether the results Experiment 1 would replicate. We found that for two of the three key dependent variables, the results of Experiment 2 were a direct replication of the results of Experiment 1. An one-way ANOVA comparing the original numerical and verbal uncertainty format with the control condition showed that format significantly affected perceived uncertainty of the numbers, $F(2, 379) = 29.57$, $p < .001$; $\eta_p^2 = .14$. Tukey's HSD post-hoc paired comparisons showed that people in the numerical uncertainty condition perceived the unemployment number to be significantly more uncertain compared to people in the control condition ($M = 4.68$ vs. 4.23, $M_{\text{diff}} = 0.45$, 95%CI[0.04;0.86], $d = 0.32$), and people in the verbal uncertainty condition perceived more uncertainty compared to both the numerical uncertainty ($M = 5.56$ vs. 4.68, $M_{\text{diff}} = 0.88$, 95%CI[0.46;1.29], $d = 0.64$) and the control conditions ($M = 5.56$ vs. 4.23, $M_{\text{diff}} = 1.33$, 95%CI[0.92;1.74], $d = 0.95$). These results are the same as the results we found in Experiment 1.

Just as in Experiment 1, an one-way ANOVA found an effect of (original) format on trust in numbers in Experiment 2, $F(2, 379) = 12.59$, $p < .001$; $\eta_p^2 = .06$. Tukey's HSD post-hoc paired comparisons showed that people in the verbal uncertainty condition perceived the number to be less reliable compared to the control condition ($M = 3.46$ vs. 4.29, $M_{\text{diff}} = -0.83$, 95%CI[-1.23;-0.43], $d = 0.61$) and the numerical uncertainty condition ($M = 3.46$ vs. 4.04, $M_{\text{diff}} = -0.58$, 95%CI[-0.98;-0.18], $d = 0.42$), with no significant difference between the control and numerical uncertainty conditions. These results replicated the results of Experiment 1.

However, whereas in Experiment 1 we found that verbal uncertainty communication reduced participants' trust in the source, this effect was not replicated in Experiment 2: an ANOVA of (original) format on trust in the source did not show a significant effect, $F(2, 379) = 2.16$, $p = .12$; $\eta_p^2 = .01$. In this Experiment, there were no significant differences in how trustworthy people perceived the source to be between uncertainty communication conditions.

Figure S2 presents the means (with 95% CIs) for all conditions of the experiment in one graph, for perceived uncertainty (Panel A), trust in the number (Panel B), and trust in the source (Panel C).

Affective responses. Participant's affective reaction to uncertainty communication was assessed with a feeling thermometer directly after the manipulation. We first tested whether the results of Experiment 1 would replicate, with a one-way ANOVA comparing the control condition with the verbal and numerical original magnitude conditions. The results showed that there was no effect of uncertainty communication on affect, $F(2, 378) = 0.08$, $p = .92$. A two-way ANOVA testing specifically for the effect of magnitude (low, original, vs high) and format (numerical vs verbal) also did not show any significant main effect of format ($F(1, 741) = 2.74$, $p = .10$), or magnitude ($F(2, 741) = 0.10$, $p = .91$), nor an interaction ($F(2, 741) = 1.22$, $p = .30$).

Feeling uncertain. Among the key dependent variables, we asked people to what extent the number made them feel uncertain. A one-way ANOVA comparing the control condition with the original formats showed that, just like in Experiment 1, verbal uncertainty communication resulted in higher levels of reported feelings of uncertainty compared to numerical ($M = 5.83$ vs. 4.83, $M_{\text{diff}} = 1.00$, 95%CI[0.46;1.54], $d = 0.47$) or no uncertainty information ($M = 5.83$ vs. 4.84, $M_{\text{diff}} = 0.99$, 95%CI[0.45;1.53], $d = 0.45$; $F(2, 379) = 8.67$, $p < .001$; $\eta_p^2 = .04$). There was no significant difference between the control condition and numerical uncertainty communication. In line with these results, a two-way ANOVA testing specifically for the effect of magnitude and format showed a main effect of format, $F(1, 741) = 32.54$, $p < .001$; $\eta_p^2 = .04$, but no main effect of magnitude ($F(2, 741) = 0.60$, $p = .55$) nor interaction ($F(2, 741) = 1.89$, $p = .15$). Across magnitudes, verbal uncertainty communication resulted in higher levels of feeling uncertain than numerical uncertainty communication ($M = 5.70$, 95%CI[5.48; 5.92] vs $M = 4.80$, 95%CI[4.58; 5.02], $d = 0.42$).

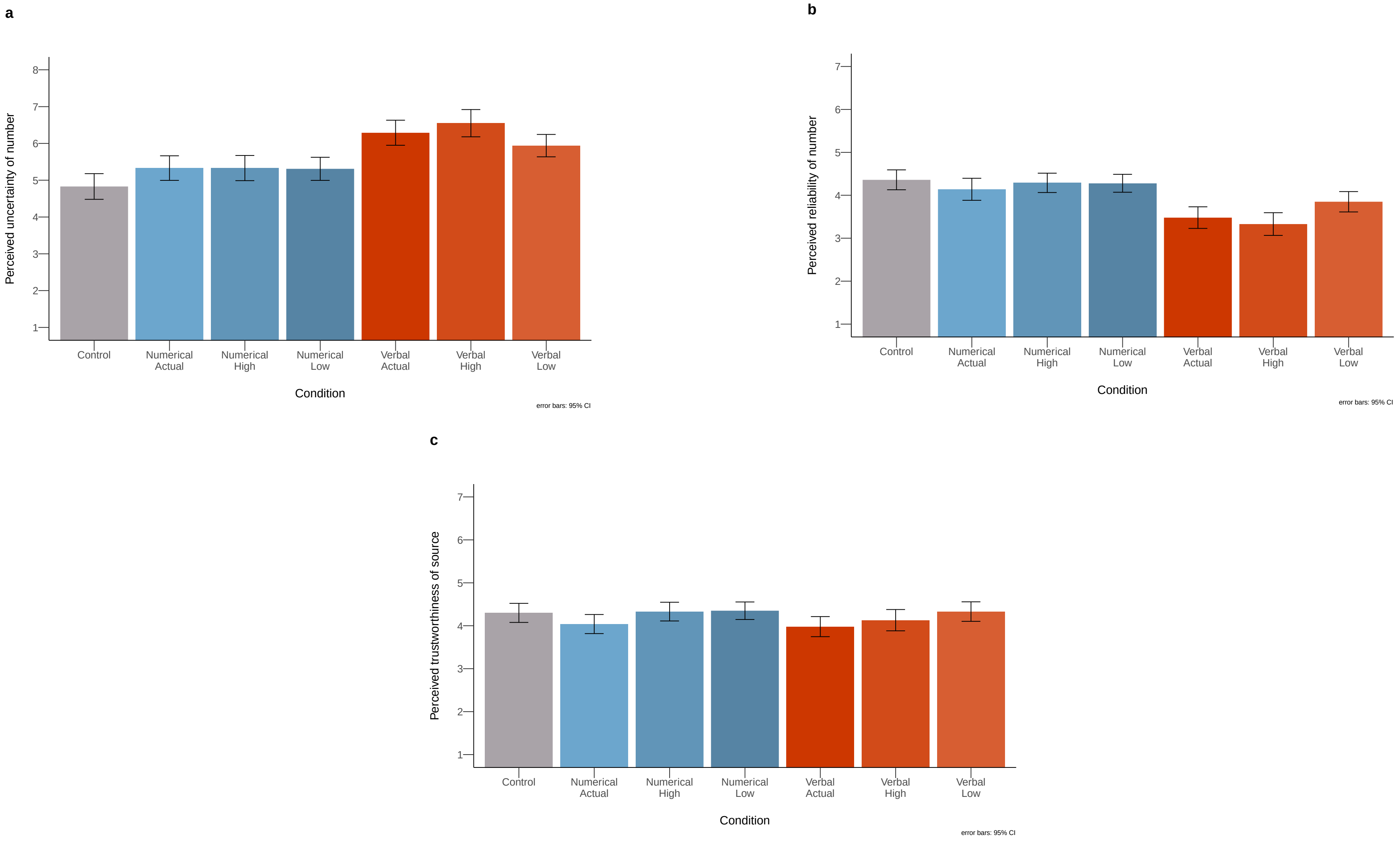

Figure S2. Means per condition for perceived uncertainty (Panel a), trust in the number (Panel b), and trust in the source (Panel c). The error bars represent 95% confidence intervals around the means.

Experiment 3

Table S9. Overview of the experimental conditions and manipulation texts of Experiment 3.

**Format**	**Experiment 3**
**Control** (no uncertainty)	**UK unemployment drops**<br>*Official figures from the first quarter of 2018 show that UK unemployment fell by 116,000 compared with the same period last year.*<br>This puts the total number of people who are unemployed at 1.42 million.<br>The number of those in work increased and wage growth improved over the same period. However, weak incomes have been a problem for a decade. "It will take a long period of wages rising above the rate of inflation for people to feel significantly better off" one economics commentator is quoted as saying.
**Numerical** range with point estimate	…by 116,000 (range between 17,000 and 215,000) …<br>…at 1.42 million (range between 1.35 and 1.49 million).
**Numerical** range without point estimate	…by between 17,000 and 215,000…<br>… at between 1.35 and 1.49 million.
**Numerical** point estimate +/- two standard errors	…by 116,000 (+/- 99,000) …<br>…unemployed at 1.42 million (+/- 70,000).
**Verbal** explicit uncertainty statement	…by 116,000 compared with the same period last year, although there is some uncertainty around this figure: it could be somewhat higher or lower.<br>…at around 1.42 million.
**Verbal** implicit uncertainty statement	…by 116,000 compared with the same period last year, although there is a range around this figure: could be somewhat higher or lower.<br>… at around 1.42 million.
**Verbal** uncertainty word	…by an estimated 116,000…<br>…at an estimated 1.42 million.
**Mixed** numerical and verbal phrase	…by an estimated 116,000 (+/- 99,000) …<br>…at around 1.42 million (+/- 70,000).

Additional Results

Affective response. Just as in Experiment 1 and 2, we assessed people's affective state with a feeling thermometer directly after the manipulation. A one-way ANOVA testing for differences between different formats of uncertainty communication showed no effect of format on people's reported affect, (F(7, 1191) = 0.80, p = .59). Again, different formats of uncertainty communication (including no uncertainty communication, the control condition) did not result in differences in how people reported the information made them feel.

Feeling uncertain. We did find differences in the extent to which people reported the numbers made them feel uncertain between the different formats. A one-way ANOVA revealed a significant effect of condition (F(7, 1191) = 7.83, p < .001; η_p^2 = .04). Tukey HSD post-hoc paired comparisons showed that compared to the control condition (M = 4.88), significantly higher levels of feeling uncertainty were reported for the "numerical +/-" condition (M = 5.60, M_{diff} = -0.72, 95%CI[-1.43;-0.01], d = 0.34), the numerical range without a point estimate (M = 5.75, M_{diff} = -0.86, 95%CI[-1.58;-0.17], d = 0.40), and the explicit verbal statement (M = 5.70, M_{diff} = -0.82, 95%CI[-1.53;-0.11], d = 0.38); the other formats did not differ significantly from the control condition.

Experiment 4

Table S10. Overview of the experimental conditions and manipulation texts of Experiment 3.

Format	Experiment 4
**Control** (no uncertainty)	**"Migration figures: EU migration still adding to UK population**<br><br>**Official figures from last year show that there were 101,000 more people coming to the UK from the EU than leaving in 2017. This is the lowest EU net migration figure since 2013, but it means that EU migrants are still adding to the UK population.**<br><br>Net migration is the difference between the number of people coming to live in the UK for at least 12 months and those emigrating. The 2017 overall net migration figure (both from the EU and non-EU countries) is also down, from record highs in 2015 and early 2016.<br><br>However, "The figures show that the government remains a long way off from meeting its objective to cut overall net migration, EU and non-EU, to the tens of thousands" one Home Affairs correspondent is quoted as saying."
**Numerical** range with point estimate	…101,000 (range between 68,000 and 132,000)…
**Numerical** point estimate +/- two standard errors	…101,000 (+/- 33,000)…
**Verbal** explicit uncertainty statement	…101,000 more people coming to the UK from the EU than leaving in 2017. The report states there is uncertainty around the exact figure - it could be higher or lower. […]
**Verbal** uncertainty word	… around 101,000…

Additional Results

Preregistered hypotheses and results. We preregistered our hypotheses for Experiment 4 on aspredicted.org (http://aspredicted.org/blind.php?x=d3xu67). This section will present each of these hypotheses in turn and discuss the level of support the experiment provides for each of the pre-registered hypotheses.

Hypothesis 1: perceived uncertainty. Based on our findings in Experiment 1 and 2, we expected that communicating uncertainty numerically (both numerical conditions) and verbally using an explicit statement would increase perceived uncertainty of the number

compared to control, with the effect for the verbal explicit uncertainty communication being larger than for numerical communication. A one-way ANOVA showed that there were significant differences between the uncertainty communication conditions (F(4, 1045) = 22.11, p < .001; η_p^2 = .08). Tukey HSD post-hoc paired comparisons showed that compared to the control condition, communicating uncertainty as a numerical range increased perceived uncertainty (M = 4.47 vs. 5.27, M_{diff} = -0.80, 95%CI[-1.22;-0.39], d = 0.50), as well as using "+/-" (M = 4.47 vs. 5.24, M_{diff} = -0.77, 95%CI[-1.19;-0.35], d = 0.50), and as an explicit verbal statement (M = 4.47 vs. 5.52, M_{diff} = -1.06, 95%CI[-1.48;-0.64], d = 0.68). In contrast to the results from Experiment 1 and 2, we find that although perceived uncertainty was descriptively higher in the explicit verbal condition (as expected), this difference was not significant compared to the numerical uncertainty conditions. The results thus were generally but not fully in line with our expectations: communicating uncertainty through numerical range, "+/- standard error", and explicit verbal statement increased perceived uncertainty, but the effect of verbal communication was not much larger than numerical communication.

Hypothesis 2: trust in numbers. We expected that communicating uncertainty numerically as "+/- standard error" and verbally using an explicit statement would decrease participants' trust in the numbers compared to the control condition; and we expected no difference between numerical range condition and control. A one-way ANOVA showed that uncertainty communication did affect trust in numbers (F(4, 1044) = 7.29, p < .001; η_p^2 = .03): the explicit verbal statement significantly reduced trust in numbers compared to the control condition (M = 3.28 vs. 3.90, M_{diff} = -0.62, 95%CI[-1.01;-0.23], d = 0.42). Both numerical formats and the verbal "around" condition did not significantly reduce trust in numbers compared to the control condition. The "+/- standard errors" format did significantly reduce trust in numbers compared to communicating the word "around" as a verbal uncertainty cue; (M = 3.57 vs. 3.97, M_{diff} = -0.40, 95%CI[-0.79;-0.002], d = 0.27), as did the explicit verbal statement (M = 3.28 vs. 3.97, M_{diff} = -0.69, 95%CI[-1.08;-0.29], d = 0.48). These results were thus again mostly but not fully in line with our hypothesis: we did find the expected reduction in trust in numbers for the explicit verbal statement compared to control, and no effect for the numerical range; but the difference between trust in numbers for the "+/- standard errors" format and control condition was not significant in this study.

Hypothesis 3: "around" as a verbal uncertainty cue. Based on our findings in Experiment 3, we expected that communicating uncertainty with the verbal cue "around" would not lead to significant differences in perceived uncertainty of the number and in trust in

the number, compared to the control condition (not communicating uncertainty at all). The results of Experiment 4 were in line with this hypothesis. There were no significant differences between communicating uncertainty with the word "around" and not communicating uncertainty at all (control condition) in perceived uncertainty of the number (around: M = 4.40, 95%CI[4.19; 4.62] vs control: M = 4.47, 95%CI[4.25; 4.68], p = .99, d = 0.04), nor trust in the number (M = 3.97, 95%CI[3.77; 4.17] vs M = 3.90, 95%CI[3.70;4.10], p = .99, d = 0.04). We interpret these findings and the findings from Experiment 3 to indicate that words like "around" and "estimated" do not effectively communicate uncertainty, as we did not find differences in perceived uncertainty of the numbers.

Hypothesis 4: trust in the source. Based on our earlier findings, we expected that communicating uncertainty (in any format tested in Experiment 4) would not lead to significant differences in trust in the source compared to not communicating uncertainty. The results of Experiment 4 support this hypothesis: we did not find a significant effect of the uncertainty communication conditions on trust in the source, assessed as trust in the civil servants who are responsible for migration statistics ($F(4, 1045) = 1.19$, p = .31).

Prior beliefs. Given the contested nature of immigration statistics, we also explored the effect of prior attitudes towards immigration on people's responses to uncertainty communication. In the main text, we report analyses based on a median split of prior attitudes towards immigration. To assess the robustness of these results we also conducted a series of hierarchical linear regressions, in which we included four dummy variables (representing the experimental conditions) at Step 1, immigration attitude as a continuous variable at Step 2, and the interaction terms between the dummies and immigration attitude at Step 3. The results were the same: for perceived uncertainty, trust in the number, and trust in the source, immigration attitude was a significant predictor (at Step 3: $\beta = -.09$, p = 0.002; $\beta = .26$, $p < .001$; $\beta = .28$, $p < .001$, respectively), but none of the interactions between immigration attitude and the experimental conditions were significant (range β = 0.02-0.17, p = .09-.83; range β = -.17 - .06, p = .09-.69; range β = -.14 - 0.06, p = .17-.67, respectively). People's prior attitudes towards immigration did influence their perceptions of uncertainty, trust in the immigration statistics, and trust in the civil servants responsible for the number, but prior attitudes did not influence how people responded to the uncertainty being communicated around the number.

Affective response. In addition, we assessed the affective response of participants to the manipulation materials with a feeling thermometer directly after the manipulation. A one-way ANOVA showed no effect of format on people's reported affect, ($F(4, 1036) = 1.05$, p =

.38): uncertainty communication did not make people feel more positive or negative about the information they read.

Feeling uncertain. The results showed that people did report differences in how uncertain the number made them feel, but only between the verbal "around" condition and the explicit verbal statement. A one-way ANOVA showed a significant effect of condition (F(4, 1045) = 4.74, p = .001; η_p^2 = .02). Tukey HSD post-hoc paired comparisons showed that this effect was driven by a difference between the explicit verbal statement and the word "around" (M = 5.99 vs. 4.99, M_{diff} = 1.00, 95%CI[0.37;1.64], d = 0.42) so that the explicit verbal statement made people feel more uncertain, with the means of all other conditions falling between these two (control: M = 5.60; numerical +/-: M = 5.55; numerical range: M = 5.57). The difference between the control condition and the word "around" was not significant, but the trend was nonetheless interesting: using the word "around" descriptively made people feel less uncertain than not communicating any uncertainty at all (M = 5.60 vs. 4.99, M_{diff} = 0.62, 95%CI[-0.02;1.26], d = 0.26).

Mediation Analyses

Although this was not the main aim of the present research, following comments from one of the reviewers, we also explored the mechanism behind people's reactions to uncertainty communication. As a first step towards this aim, we conducted exploratory mediation analyses to examine whether the effect of being exposed to uncertainty communication on people's trust in the number was mediated by people's perceptions of uncertainty. We ran mediation models using the PROCESS macro for SPSS (5) with 10,000 bootstrap samples.

For the effect of communicating uncertainty (as a numeric range, numeric +/-, and explicit verbal statements) compared to not communicating uncertainty (control condition) on trust in the number, the analysis showed evidence for full mediation through perceived uncertainty of the number (see Figure S3). The indirect effect of uncertainty communication on trust in the number (via perceived uncertainty) was -.50, Bias Corrected 95% Confidence Interval (CI)[-.65; -.35].

To further examine this mediation effect, we tested it specifically for two formats of uncertainty communication: as a numeric range (vs. control) and as an explicit verbal statement (vs. control). While the total effect of communicating a numeric range (vs. control) on trust in the number was not significant, the results presented in Figure S4 did indicate

evidence of mediation: the indirect effect via perceived uncertainty of the number was significant, -.49, 95%CI[-.68; -.30]. When uncertainty was communicated as an explicit verbal statement, there was again evidence for full mediation of perceived uncertainty of the number (see Figure S5): the indirect effect via perceived uncertainty of the number was -.63, 95%CI[-.84;-.44].

In sum, these results indicate that in the aggregate, there is a negative effect of uncertainty communication on trust in the number because people perceive more uncertainty following its communication.

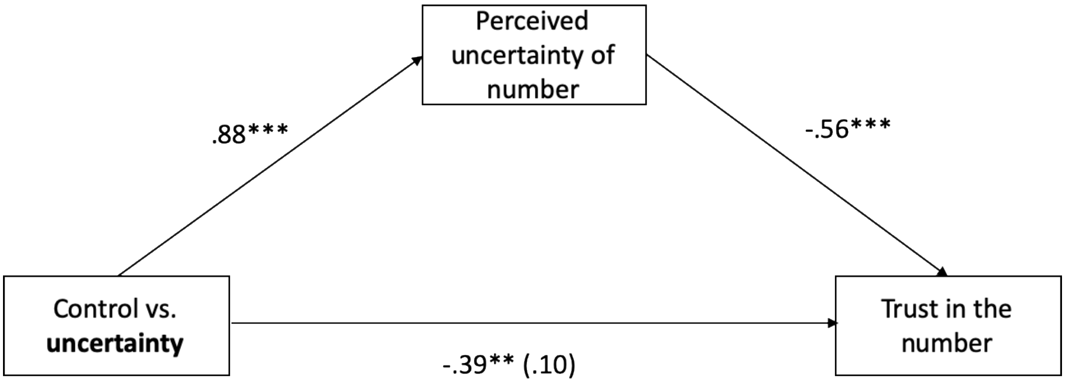

Figure S3. Results of the mediation analysis of the effect of uncertainty communication (vs. control) on trust in the number via perceived uncertainty. Regression coefficients are presented, with the direct effect of uncertainty communication on trust in the number displayed in parentheses. *$p < 0.05$, **$p < 0.01$, ***$p < 0.001$.

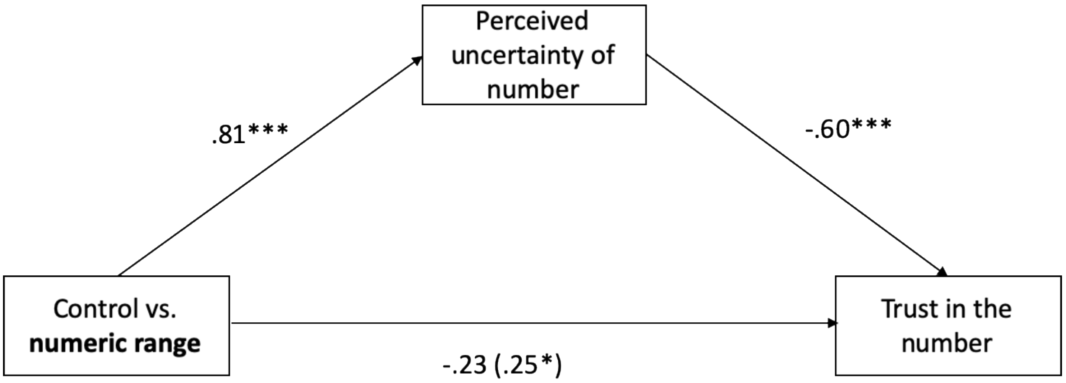

Figure S4. Results of the mediation analysis of the effect of uncertainty communication as a numeric range (vs. control) on trust in the number via perceived uncertainty. Regression coefficients are presented, with the direct effect of uncertainty communication on trust in the number displayed in parentheses. *$p < 0.05$, **$p < 0.01$, ***$p < 0.001$.

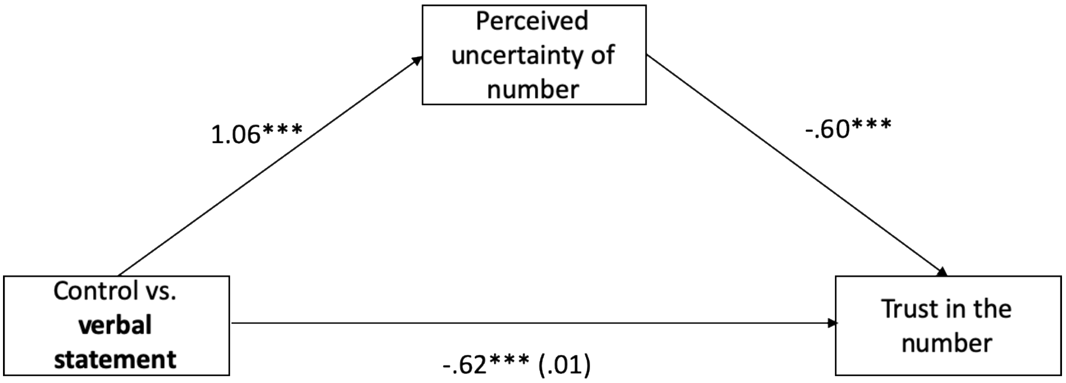

Figure S5. Results of the mediation analysis of the effect of uncertainty communication as a verbal statement (vs. control) on trust in the number via perceived uncertainty. Regression coefficients are presented, with the direct effect of uncertainty communication on trust in the number displayed in parentheses. *$p < 0.05$, **$p < 0.01$, ***$p < 0.001$.

Experiment 5: Field experiment

Pilot study

We conducted a pilot study on the live BBC News website to gain experience with the technical and journalistic context of the field experiment, in collaboration with BBC News online. On the 10th of September 2019, we used the ONS' release of the Labour Market Statistics bulletin as the context for the pilot study. The journalistic team for wrote a news article based on the ONS release with a focus on wage growth, and we worked with the journalistic team and Head of Statistics to select a target figure to communicate uncertainty around before the news article was published on the website. For this pilot study, that was earning growth (excluding bonuses) and was the first figure communicated in the article: "Earnings excluding bonuses grew at an estimated annual pace of 3.8% (between 3.3% and 4.3%) in the May to July period, down slightly from the previous reading." The pilot study had the same design as the field experiment: readers of the website were shown one of three versions of the article, either without additional uncertainty (control condition), with uncertainty communicated as a verbal cue ("estimated 3.8%") or with uncertainty communicated as a numerical range ("3.8% (between 3.3% and 4.3%)"). Participants were asked the same questions as reported for Experiment 5, adapted for this target number ("the earnings figure in the story").

Because this was a pilot study, we decided to start the experiment after the first release in the first update of the story, which meant that we missed much of the morning traffic on the website. In addition, technical issues meant that all users of the BBC News mobile application were shown only the control condition; the experimental conditions were only shown to users of the website in a computer browser. We ended data collection after 48 hours with a total of 589 participants: 366 in the control condition, 115 in the numeric condition, and 108 in the verbal condition. Total sample consists of 503 male and 70 female participants (2 other, 14 missing), with a mean age of 49.16 (*SD* = 15.41). Education level was distributed as follows: No school = 1.4%, School = 26.5%, Bachelor degree = 37%, Higher degree = 33.4%.

Results. A series of ANOVAs showed no significant differences between uncertainty communication conditions for any of the outcomes.

Pilot experiment news article text (first paragraph):

Wage growth stays strong as unemployment falls

Wages have continued to grow at a strong pace and employment remains at record highs, official figures show.

Earnings excluding bonuses grew at an estimated annual pace of 3.8% (between 3.3% and 4.3%) in the May to July period, down slightly from the previous reading.

Including bonuses, wages rose at an annual pace of 4% - the highest rate since mid-2008.

The unemployment rate dipped to 3.8%, while the estimated employment rate remained at a record 76.1%.

Click here to take part in a short study about this article run by the University of Cambridge.

Additional Results

In addition to the measures described in the main text, participants answered a comprehension question ("How did the unemployment rate figure change in the June to August period, compared to the previous period?" from 1 = fallen a lot, 2 = fallen a little, 3 = not really changed, 4 = gone up a little, and 5 = gone up a lot). A one-way ANOVA showed no significant effect of uncertainty communication ($F(2, 1525) = 0.79$, $p = .46$). The distribution of answered showed that most participants answered in line with the content of the news story that unemployment had gone up a little (58.7%), a sizable minority answered it had fallen a little (28.3%), whereas the other answers were selected less frequently (gone up a lot: 2.7%, not really changed: 8%, fallen a lot: 2.1%).

References

1. European Social Survey, "ESS Round 8 Source Questionnaire" (2016).
2. A. M. van der Bles, T. Postmes, B. LeKander-Kanis, S. Otjes, The Consequences of Collective Discontent: A New Measure of Zeitgeist Predicts Voting for Extreme Parties. *Polit. Psychol.* **39**, 381–398 (2018).
3. S. van der Linden, A. Leiserowitz, S. Rosenthal, E. Maibach, Inoculating the Public against Misinformation about Climate Change. *Glob. Challenges* **1**, 1600008 (2017).
4. E. R. Thompson, Development and Validation of an Internationally Reliable Short-Form of the Positive and Negative Affect Schedule (PANAS). *J. Cross. Cult. Psychol.* **38**, 227–242 (2007).
5. A. F. Hayes, *Introduction to mediation, moderation, and conditional process analysis: A regression-based approach* (Guilford Press, 2013).
